# Identification of *in planta* bioprotectants against Fusarium wilt in *Medicago sativa* L. (lucerne) from a collection of bacterial isolates derived from *Medicago* seeds

**DOI:** 10.3389/fmicb.2025.1544521

**Published:** 2025-02-26

**Authors:** Shenali Subodha Herath Dissanayakalage, Jatinder Kaur, Saidi R. Achari, Timothy I. Sawbridge

**Affiliations:** ^1^Agriculture Victoria, AgriBio Centre for AgriBioscience, Bundoora, VIC, Australia; ^2^School of Applied Systems Biology, La Trobe University, Bundoora, VIC, Australia

**Keywords:** Fusarium wilt, *Medicago* seeds, lucerne, alfalfa, *Pseudomonas* sp., *Paenibacillus* sp., biocontrol, plant pathogens

## Abstract

Fusarium wilt caused by *Fusarium oxysporum* f. sp. *medicaginis* (Fom) is an important disease affecting lucerne/alfalfa cultivations worldwide. *Medicago sativa* L. (lucerne) is one of the major legume crops in global forage industry. This study aimed to identify bacteria capable of biologically controlling the wilt pathogen through a comprehensive screening of bacterial isolates obtained from domesticated and wild growing *Medicago* seeds. Using a multi-tiered evaluation pipeline, including *in vitro*, soil-free and potting mix-based pathogenicity and bioprotection assay systems, the bioprotection efficacy of 34 bacterial isolates derived from *Medicago* seeds was initially evaluated against six *Fusarium* strains *in vitro*. *Fusarium oxysporum* (Fo) F5189, which has previously been characterized as a *Fusarium oxysporum* f. sp. *medicaginis* isolate causing *Fusarium* wilt in lucerne was selected for *in planta* assays. Lucerne cultivars Grazer and Sequel, representing susceptible and resistant genotypes were chosen to assess the disease progression. Pathogenicity and bioprotection time-course studies were conducted to understand the temporal dynamics of host-pathogen interactions and efficacy of the bioprotectants. The disease symptoms were scored using a disease rating index developed in this study. The results indicated variability in bioprotection efficacy across bacterial isolates, with some strains suppressing disease in both soil-free and potting mix-based systems. *Paenibacillus* sp. (Lu_MgY_007; NCBI: PQ756884) and *Pseudomonas* sp. (Lu_LA164_018; NCBI: PQ756887) were identified as promising bioprotectants against Fusarium wilt under tested growth conditions. The time-course studies highlighted the critical role of persistent biocontrol activity and precise timing of biocontrol application for achieving long-term disease suppression. Overall, the observed reduction in disease severity underscores the potential of these bioprotectants as sustainable strategies for managing Fusarium wilt in lucerne cultivars. However, comprehensive molecular-level analyses are warranted to elucidate the underlying pathogenicity and bioprotection mechanisms, offering valuable insights for the development of more precise and effective future biocontrol strategies in agricultural systems.

## 1 Introduction

Lucerne or alfalfa (*Medicago sativa* L), often referred to as ‘queen of forages’ is a significant perennial pasture legume, cultivated across 30 million hectares worldwide (Adhilakshmi et al., 2008; Annicchiarico, 2015; Zhang et al., 2024). Its high nutritional content, significant biomass production, nitrogen fixing ability and adaptability to hostile environments have strengthened its position as the most widely used forage crop (Bingham et al., 1994; Teixeira et al., 2023; Zhang et al., 2024). However, lucerne cultivation is severely constrained by soil-borne fungal pathogens, particularly species within the genus *Fusarium*. The genus *Fusarium* (Sordariomycetes: Hypocreales: Nectriaceae) is composed of ascomycete fungi that includes putatively non-pathogenic and pathogenic strains, collectively responsible for considerable yield losses in lucerne cultivation (Krnjaja et al., 2003; Okungbowa and Shittu, 2012; Achari et al., 2021; Abbas et al., 2022). Among the pathogenic strains is, *F. proliferatum,* (Fp) one of the main pathogens causing Fusarium root rot, a serious soil-borne disease that compromises root health and reduces crop productivity (Cong et al., 2016; Wang et al., 2023). Fusarium wilt is another economically important vascular wilt disease caused by a root-borne fungal pathogen, Fom (Jones and Weimer, 1928) W.C. Snyder and H. N. Hans. This disease is characterized by foliar wilting and yellowing, stunted growth, reddish discolouration of the xylem vessel and in severe cases, plant death (Antonopoulos and Elena, 2008; Sampaio et al., 2020). The accumulation of plant defence substances such as tyloses, gums and phenolic compounds, in response to Fom infection, can impede pathogen systemic dissemination but may also block the xylem tissue, hindering water transport and aggravating wilt symptoms (Kashyap et al., 2020; Jangir et al., 2021). This pathogen, Fo, is extremely difficult to control, as its spores are highly resilient, capable of surviving in soil for decades, even in the absence of the host plants (Blok and Bollen, 1996; Thatcher et al., 2016). As a result, conventional management strategies for Fusarium wilt rely heavily on systemic fungicides such as carbendazim and soil fumigants such as dazomet, chloropicrin and 1,3-dichloropropene (Antonopoulos and Elena, 2008; Zhao et al., 2016; Sampaio et al., 2020). However, excessive usage of these fungicides has led to the development of fungicide-resistance in pathogens, detrimental effects on non-target microbes, environmental issues and risks to human health (Yadeta and Thomma, 2013; Dukare et al., 2019). Therefore, it is critical to explore alternative approaches for sustainable and eco-safe management of soil-borne pathogens in the lucerne cultivation.

Biological control, in particular, has emerged as a promising alternative to conventional disease management strategies, offering the ability to enhance plant resilience while maintaining ecological balance (Dukare et al., 2022). Pal and Gardener (2006) has defined biological control as “purposeful utilization of introduced or resident living organisms, other than disease resistant host plants, to suppress the activities and populations of one or more plant pathogens.” The biological control of fungal phytopathogens by beneficial microbes encompasses various mechanisms, including the production of antifungal compounds such as hydrolytic enzymes, antibiotics, phytohormones, hydrogen cyanide and volatile metabolites, as well as competition for nutrient resources, which collectively reduces disease incidence (Tari and Anderson, 1988; Voisard et al., 1989; Palumbo et al., 2005; Defago and Haas, 2017; Dehbi et al., 2023; Egamberdieva et al., 2023).

In recent years, bacterial endophytes have gained significant attention as *in planta* bioprotectants due to their unique attributes compared to fungal endophytes. These beneficial bacterial endophytes rapidly colonize plant tissues (Walitang et al., 2017; Tufail et al., 2022), produce broad-spectrum metabolites (Keswani et al., 2020; Salazar et al., 2023) and serve a dual role in biocontrol and plant growth promotion (Kandalepas et al., 2015), making them highly effective and practical in agricultural applications. For instance, endophytic plant growth promoting rhizobateria (PGPR) can induce systemic resistance [mediated by jasmonic acid (JA) and ethylene] in the host plant which can enhance the host defence against the subsequent infection (Peer, 1991; Pieterse et al., 1998; van Loon et al., 1998; Pieterse et al., 2014). Additionally, bacterial endophytes exhibit a lower risk of transitioning from mutualistic to pathogenic behaviour under stress conditions, ensuring greater reliability in field applications (Miliute et al., 2015; Ganie et al., 2022). This study specifically focuses on beneficial bacterial endophytes from the *Medicago* seed microbiome as potential biocontrol agents against Fusarium wilt. The elicitation of bioprotection by bacterial seed endophytes has received increasing attention due to their efficacy and suitability for field applications (Díaz Herrera et al., 2016; Li et al., 2020; Wang et al., 2022). Several seed endophytes have been found to offer bioprotection by producing lipopeptides such as mycobacillin, iturin and surfactin (Gagne-Bourgue et al., 2013). For instance, some strains of *Pseudomonas* spp. and *Bacillus* spp. have been well documented for their biocontrol activity against *F. oxysporum* f. sp. *lycopersici*, the causal agent of tomato wilt (Sundaramoorthy and Balabaskar, 2013). Similarly, endophytic *Enterobacter* strains isolated from rice have demonstrated biocontrol activity against various phytopathogens through the production of volatile anti-fungal compounds (Mukhopadhyay et al., 1996). However, efficacy of the bioprotectants is heavily influenced by the host genotype, bacterial strain, biotic and abiotic factors such as temperature and moisture as well as disease progression stage (Hannusch and Boland, 1996; Boland, 1997; Smith et al., 1999; Kazmar et al., 2000; Bonaterra et al., 2022). This emphasises the need for comprehensive screening and evaluation of bacterial strains to determine the most potent candidates. Despite growing research, there is a gap in the literature in understanding specific interactions among bioprotectants, host plant and the target pathogen during an infection. Our study aims to investigate some of these interactions by implementing a time-course study, as the timing of specific interactions is crucial for understanding temporal dynamics and progression of a disease. We aim to systematically evaluate bacterial isolates from seeds of domesticated and wild growing *Medicago* spp. using a three-tiered evaluation pipeline to identify potential bioprotective agents effective in controlling Fusarium wilt pathogens in lucerne cultivars under commercial settings.

## 2 Materials and methods

### 2.1 Isolation of bacterial strains from *Medicago* seeds

Nine different domesticated lucerne seed accessions were obtained from various seed companies across Australia. Additionally, 10 *Medicago* crop wild relative (CWR) seed accessions were sourced from Australian Pasture GenBank (APG; Supplementary Table S1). The seeds were washed by rinsing with autoclaved RO (reverse osmosis) water for four times followed by germination under sterile conditions (on wet sterile filter papers in sealed 90 mm petri dishes) for 7 days. Seed germination rates were assessed prior to bacterial isolations (Supplementary Table S1) and 20 seedlings from each cultivar were selected after 7 days of germination. The selected seedlings were suspended in 300 μl of sterile 1 × phosphate buffer saline (PBS) and ground using a Qiagen Tissue Lyser II (2 × 30 s at 25 Hz). The ground seedlings were centrifuged at 6,000 revolutions per minute (rpm) for 5 min. The supernatant from each cultivar was serially diluted in PBS (1:10, 100 μl in 900 μl) and plated onto Reasoner’s 2A agar (R2A) to isolate pure distinct bacterial colonies from 10^−2^ to 10^−5^ dilutions. Bacterial pure cultures in nutrient broth (NB, BD Bioscience) were stored at −80°C in 20% glycerol (v/v) until further use. A total of 530 unique bacterial isolates were obtained. The full length 16S rRNA gene sequencing was performed on 324 of the 530 isolates using Sanger sequencing. The 16S amplicon raw data of the isolates was processed using the program Geneious Prime (version 2024.0.5; Biomatters Ltd., Auckland, New Zealand) and taxonomic identification was carried out through NCBI BLAST. A total of 34 bacterial isolates were chosen from the 324 identified isolates to assess their potential biological control attributes against Fusarium wilt pathogen (The taxonomic information and rationale for the selection process is summarized in Supplementary Table S2; Herath Dissanayakalage et al., 2025, manuscript in preparation).

### 2.2 Pathogen strains

The *Fusarium* isolates used in this study were obtained from Victorian Plant Pathogen Herbarium (VPRI, Bundoora, VIC, Australia). These isolates included, three isolates each of Fp (42191, 42409 and 42958) and Fo (44256, 44314 and 44257) species (Supplementary Table S3). To maintain the viability and purity, all *Fusarium* strains were cultured on ½ potato dextrose agar (½ PDA, Oxoid or Amyl Media, Australia) at room temperature in the dark. The pure cultures in nutrient broth were stored at −80°C in 15% glycerol (v/v).

### 2.3 *In vitro* screening of the biocontrol effect of potential bacterial strains against *Fusarium* pathogens

**Figure 1:**
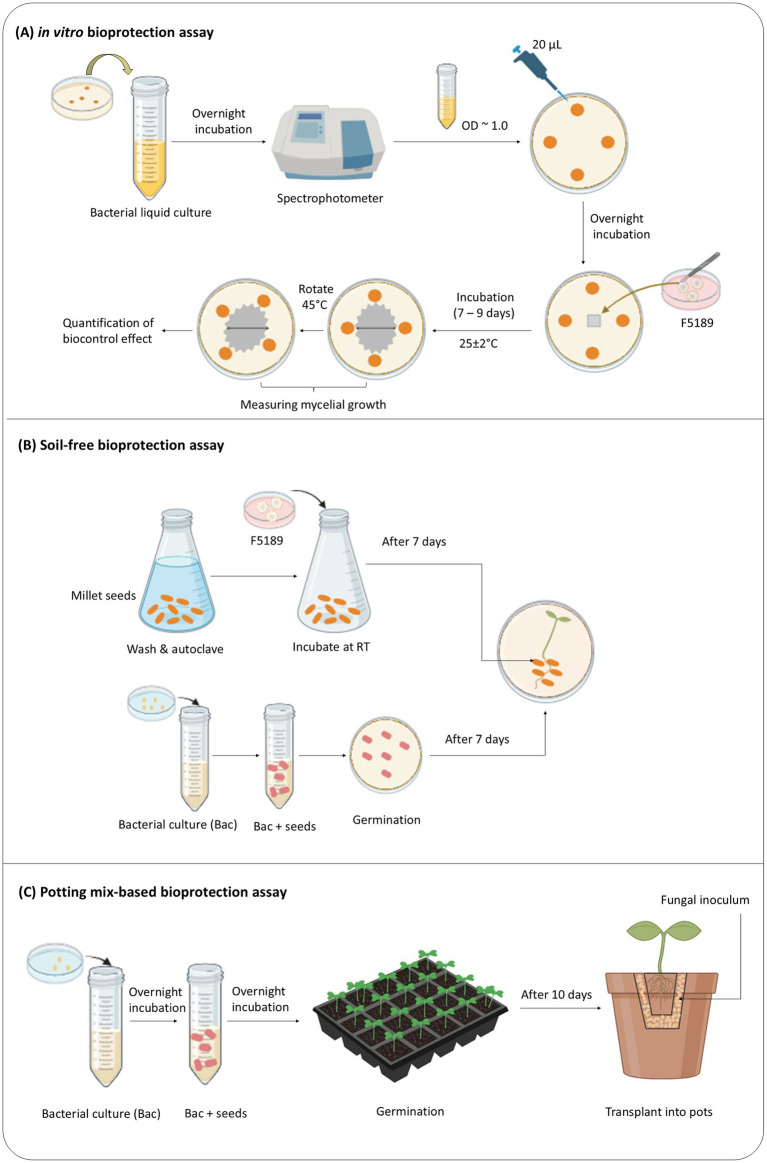
**(A-C)** Schematic overview of the three-tiered bioprotection assay pipeline, from *in vitro* screening to *in planta* evaluation.

A dual-culture *in vitro* assay was designed to assess the ability of 34 bacterial strains to supress or inhibit the growth of six isolates of *Fusarium* phytopathogens (Figure 1A). Bacterial strains were cultured in NB overnight and their concentrations were adjusted to OD_600_ ~ 1.0 (10^8^–10^9^ CFU/ml). Twenty microlitres (20 μl) of bacterial suspension was drop inoculated at four equidistant points on nutrient agar (NA, BD Bioscience) plate, which was equally spaced from the centre. The plate was incubated overnight at 25 ± 2°C. Then, a 6 × 6 mm plug of the pathogen strain was taken from the edge of the actively growing fungal hyphae on a ½ PDA plate and placed at the centre of each NA plate containing drop inoculated bacteria (Li et al., 2020). The NA plates drop inoculated with NB and a plug of *Fusarium* strain at the centre were considered as pathogen controls. All bioassay plates were incubated at 25 ± 2°C for 7 to 9 days to accommodate differential growth rates of the *Fusarium* pathogens. Inhibition of *Fusarium* pathogens were measured after designated incubation periods by measuring the mycelium radius twice. One reading was taken through the centre of the mycelium from one bacterial inoculation point to the other, and the other reading was taken by rotating the bioassay plate 45° from the first measurement. The formula of Vincent (Singh et al., 2011) was used to calculate the percentage inhibition:


I=C−T/C×100

Where, I is percentage inhibition, C is the radial mycelial growth of the control (mm) and T is the radial mycelial growth of the pathogen in the dual culture. As biological replicates, three plates were prepared for each treatment. Sterile nutrient broth was used as the inactive control to replace bacteria. For statistical analysis, One-way ANOVA and Tukey test were conducted using OriginPro 2020 (version 9.7.0.188 [Academic]) for any significant difference (*p* < 0.05) among the treatments. Of these 34 bacterial strains, two strains were selected based on their *in vitro* bioprotection activity for *in planta* bioassay screenings.

### 2.4 Soil-free *in planta* screening of candidate bacterial strains for their potential biocontrol activity against *Fusarium* pathogens

#### 2.4.1 Selection of *Fusarium* strain for *in planta* bioprotection assay

The selection of a suitable Fo isolate for the *in planta* bioprotection assays was based on its pathogenicity and relevance to *M. sativa*. Preliminary *in vitro* screenings confirmed that all three Fo. isolates tested were pathogenic to *M. sativa,* sharing similar ecological niches and infection mechanisms. Out of these three, two of the Fo isolates (44256, 44257) have shown stronger inhibition in the presence of the two selected candidate bioprotectant bacterial strains selected for the study. To ensure experimental integrity and minimize bias, isolate 44257 (herein after referred to as F5189 [strain ID]) was randomly selected for the *in planta* evaluation. The F5189 fungal inoculum was prepared using sterile millet seeds (Noor et al., 2022; Figure 1B; Refer to Supplementary section 1 for the detailed protocol of fungal inoculum preparation).

#### 2.4.2 Lucerne seedling preparation

Eighteen commercial lucerne seed accessions, including those used for bacterial isolations, were obtained from various seed companies across Australia to set up the soil-free assay (Supplementary Table S1). The seeds were rinsed four times with autoclaved RO water and germinated on wet sterile filter paper in sealed 90 mm petri dishes under sterile conditions with ample light conditions at 25 ± 2°C for 7 days. Healthy seedlings with fully grown cotyledon leaves and 5–7 cm in length were selected for the assays. Seedlings with noticeable blackening at root-shoot junction (RSJ), which was likely caused by seedborne pathogens were eliminated from the study.

#### 2.4.3 Design and implementation of *Fusarium* soil-free *in planta* pathogenicity assay

A pathogenicity assay was designed to establish baseline effect of F5189 on lucerne plants. Selected healthy lucerne seedlings were placed on new petri dishes (1 seedling/petri-dish) with wet, sterile filter papers. The seedling roots were covered with approximately 2 g of *Fusarium*-millet grain inoculum. Autoclaved millet grains without *Fusarium* inoculum were used as the negative control (Figure 1B). The assay plates were placed on laboratory bench under ambient light and controlled laboratory temperature (23 ± 2°C). The seedlings were monitored daily for 14 days, and disease symptoms were rated at four time points (3 dpi [days post inoculation], 6, 8 and 14 dpi), using a disease scoring system developed during this study (Refer to Supplementary section 2 for a detailed description of the disease scoring index), based on visual assessment principles informed by previous methodologies for other crop-pathogen systems (Xu et al., 2004; Murty and Shim, 2021) This scoring system is novel and specifically tailored for evaluating Fusarium wilt in lucerne cultivars under soil-free conditions. Five replicates were used for each treatment group: lucerne seedlings with *Fusarium*-millet grain inoculum and lucerne seedlings with autoclaved millet grains. For statistical analysis, One-way ANOVA and Tukey test were conducted using OriginPro 2024. Based on the disease index corresponding to each lucerne cultivar included in the pathogenicity assay, the most susceptible and the most resistant cultivars were identified for the soil-free *in planta* bioprotection assay.

#### 2.4.4 Soil-free *in planta* bioprotection assay design

A similar experimental setup used in the soil-free *in planta pathogenicity* assay was used for soil free *in planta* bioprotection assay with following modifications. The selected bacterial strains were grown in NB overnight at 28 ± 1°C. Then, their concentrations were adjusted to OD_600_ = ~ 0.5 (Li et al., 2020). The washed seeds were imbibed in concentration-adjusted bacterial liquid cultures overnight at 28 ± 1°C (Figure 1B). Subsequently, seeds were germinated as per section 2.4.2. The *in planta* bioprotection assay was performed as per section 2.4.3. The seedlings were monitored daily for 14 days, and disease symptoms were scored at six time points: 3, 5, 7, 10, 12, and 14 dpi.

### 2.5 *In planta* screening of candidate bacterial strains for their potential biocontrol activity against *Fusarium* pathogens in potting mix

#### 2.5.1 Design and implementation of *in planta* pathogenicity assay in potting mix

The inoculated and uninoculated lucerne seeds were prepared as described for the soil-free assay and germinated in seedling trays filled with standard potting mix for 10 days. The potting mix (Australian Growing Solutions Pty. Ltd., Tyabb, Victoria, Australia) consisted of a mixture of saw dust, propagation sand, gypsum, pine bark, and a soil wetting agent (SaturAid®, Debco Pty. Ltd., Tyabb, Victoria, Australia). The seedlings were grown in growth cabinets with 16 h of light and 8 h of dark at 25 and 20°C, which have been optimized for consistent *M. sativa* seedling development. After 10 days, healthy seedlings were selected for the *in planta* assay. The selected seedlings were transplanted as a plug from the seedling tray into 90 mm pots with plug-shaped holes (30 mm × 30 mm × 50 mm) lined with *Fusarium*-millet grain inoculum. Control seedlings were transplanted into the pots with autoclaved millet grains lined plug-shaped holes. The transplanted seedlings were further grown in pots for 14 days with daily monitoring for disease symptom development. Disease ratings were performed at 3, 5, 7, 10 and 14 dpi, using a previously developed rating scale specifically designed to assess Fusarium wilt in lucerne cultivars under potting mix-based conditions (Refer to Supplementary section 3 for a detailed description of the disease scoring index). For statistical analysis, One-way ANOVA and Tukey test were conducted using OriginPro 2024.

#### 2.5.2 *In planta* bioprotection assay in potting mix

The *in planta* bioprotection assay was performed on Grazer, the most susceptible lucerne cultivar (Figure 1C). The seeds were treated as per section 2.4.2. Seedling preparation, *in planta* assay and disease rating were carried out as per section 2.5.1. The disease rating was performed at 3, 5, 7, 10, and 14 dpi. After 14 days the seedlings were uprooted and root and shoot measurements were taken.

## 3 Results

### 3.1 *In vitro* screening of the biocontrol effect of potential bacterial strains against *Fusarium* pathogens

The *in vitro* bioprotection activity of the 34 bacterial strains was tested against three isolates each of Fp. (Supplementary Figure S1) and Fo. (Supplementary Figure S2) phytopathogens. Of the 34 bacterial strains assessed, three of the *Pseudomonas* spp. strains: Lu_LA164_018, Lu_F5_006 and Lu_F5_029 have shown biocontrol effects against all the six isolates of *Fusarium* spp. (Table 1). However, only six of the 34 bacterial strains, including one each of *Duffyella gerundensis* (Lu_F5_028), *Paenibacillus* sp. (Lu_MgY_007) as well as four strains of *Pseudomonas* sp. (Lu_F5_029, Lu_F5_006, Lu_TR935_014 and Lu_LA164-018) have shown biosupression against all the three isolates of Fp. screened. Moreover, seven bacterial strains, including one each from *Enterobacter* sp. (Lu_Au_053), *Pseudomonas* sp. (Lu_TR758_011) and *Pantoea* sp. (Lu_TR771_006) along with four strains from Enterobacteriaceae (Lu_LA841_009, Lu_TR758_007, Lu_LT198_018 and Lu_LT198_002) have demonstrated selective bioprotection activity by effectively reducing fungal radial growth of the three isolates of Fo., but showed no biocontrol activity against any of the strains of Fp. tested. Significant fungal radial growth reduction was observed in some dual cultures prepared with latter strains. For example, Lu_LA164_018 has reduced the growth Fp. 42409 and Fo. 44256 by 59.1 and 66.1% respectively, compared to the blank control. Furthermore, Lu_MgY_007 has demonstrated a mycelial radial growth reduction across four strains of *Fusarium* spp. ranging from 29.4 to 50.2%. A 50.4% fungal radial growth reduction was also observed in the dual culture of Lu_F5_006 with Fp. 42409. In contrast, three strains of *Paenibacillus* sp. (Lu_Sv_042 [NCBI: PQ756886], Lu_LT198_042 and Lu_TR771_007, along with one strain belonging to *Duganella* sp. (TR935-010 [NCBI: PQ756910]) have shown no biocontrol activity against any of the six strains of *Fusarium* spp. pathogens. These findings revealed the variability of bioprotection efficacy among the 34 bacterial strains, with some strains indicating broad-spectrum bioprotection activity, while other strains were species-specific or inactive against all tested *Fusarium* spp.

**Table 1:** Percentage inhibitions of mycelial growth of *Fusarium* spp. by potential bacterial candidates in *in vitro* dual cultures.

Bacterial isolate ID	Host plant	Closest 16S rRNA sequence match (NCBI BLAST)	Percentage inhibition of mycelial growth					
			*Fusarium proliferatum*			*Fusarium oxysporum*		
			42191	42409	42958	44257 (5189)	44256 (5190)	44314
Lu_MgY_007	*M. sativa*	*Paenibacillus terrae*	35.9	50.2	29.4	48.8	50.2	0.0
Lu_R6_023		*Duffyella gerundensis*	27.9	0.0	0.0	25.7	0.0	0.0
Lu_Sv_042		*Paenibacillus* sp. FSL E2-0151	0.0	0.0	0.0	0.0	0.0	0.0
Lu_Au_053		*Enterobacter kobei*	0.0	0.0	0.0	26.9	21.7	24.4
Lu_Au_058		*Enterobacter* sp. MLB27	0.0	24.3	0.0	15.3	0.0	25.8
Lu_F5_006		*Pseudomonas koreensis*	31.4	50.4	9.9	25.5	25.3	35.1
Lu_F5_008		*Massilia* sp. *4D10*	27.1	0.0	0.0	0.0	0.0	8.2
Lu_F5_028		*Duffyella gerundensis*	27.1	27.0	10.1	22.7	0.0	0.0
Lu_F5_029		*Pseudomonas* sp. XBBSY4	32.2	24.6	10.1	28.1	33.3	29.0
Lu_LA164_003	*M. laciniata*	*Pantoea agglomerans*	26.5	0.0	0.0	26.5	23.6	26.7
Lu_LA164_009		*Duganella* sp. PH3	29.8	0.0	0.0	0.0	0.0	0.0
Lu_LA164_012		*Duganella* sp. PH3	0.0	25.0	0.0	0.0	0.0	0.0
Lu_LA164_018		*Pseudomonas* sp. R11-45-07	38.6	59.1	32.0	34.7	66.1	42.3
Lu_LA700_W009		*Pantoea* sp.	0.0	28.8	0.0	23.9	0.0	24.2
Lu_LA841_007		Enterobacteriaceae bacterium SAP758.2	29.8	27.2	0.0	25.3	25.1	25.6
Lu_LA841_009		Enterobacteriaceae bacterium SAP758.2	0.0	0.0	0.0	27.7	21.5	29.2
Lu_LA841_015		*Pseudomonas* sp. Na1	34.5	27.9	0.0	0.0	31.7	0.0
Lu_LT177_010	*M. littoralis*	*Paenibacillus xylanexedens*	26.1	27.9	0.0	13.1	0.0	0.0
Lu_LT198_002		Enterobacteriaceae bacterium SAP758.2	0.0	0.0	0.0	24.9	21.5	22.4
Lu_LT198_003		*Pantoea agglomerans*	27.9	0.0	0.0	0.0	19.1	0.0
Lu_LT198_010		*Pseudomonas ovata*	0.0	25.9	0.0	27.1	22.4	29.6
Lu_LT198_018		Enterobacteriaceae bacterium SAP758.2	0.0	0.0	0.0	12.1	25.9	19.0
Lu_LT198_042		*Paenibacillus nicotianae*	0.0	0.0	0.0	0.0	0.0	0.0
Lu_LT198_W003		*Pseudomonas lutea*	26.3	25.4	0.0	28.3	23.6	0.0
Lu_LT235_004		*Pantoea agglomerans*	0.0	0.0	0.0	25.5	0.0	29.2
Lu_TR758_007	*M. truncatula*	Enterobacteriaceae bacterium SAP758.2	0.0	0.0	0.0	25.1	26.3	29.9
Lu_TR758_008		*Paenibacillus* sp. 3Cp1	0.0	0.0	0.0	0.0	0.0	7.5
Lu_TR758_011		*Pseudomonas* sp. SAP829.3	0.0	0.0	0.0	26.9	25.1	25.6
Lu_TR758_015		Enterobacteriaceae bacterium SAP758.2	0.0	30.1	9.9	24.9	21.5	27.2
Lu_TR758_W005		*Pantoea eucalypti*	0.0	0.0	0.0	26.3	0.0	29.2
Lu_TR771_006		*Pantoea agglomerans*	0.0	0.0	0.0	26.5	20.3	29.0
Lu_TR771_007		*Paenibacillus* sp. 3Cp1	0.0	0.0	0.0	0.0	0.0	0.0
Lu_TR935_010		*Duganella* sp. PH3	0.0	0.0	0.0	0.0	0.0	0.0
Lu_TR935_014		*Pseudomonas* sp.	34.9	29.2	22.2	28.3	36.2	0.0

#### 3.1.1 Selection of bacterial strains for *in planta* bioprotection assay

Of the 34 bacterial strains assessed for *in vitro* bioprotection activity against *Fusarium* pathogens, four of the bacterial strains; one each from *Pseudomonas* sp. strain (Lu_LA164_018) and *Duganella* sp. strain (Lu_TR935_010) and two from *Paenibacillus* sp. strains (Lu_MgY_007 and Lu_Sv_042) were chosen for *in planta* bioprotection assay. Preliminary *in vitro* screening has identified Lu_LA164_018 and Lu_MgY_007 strains as the most effective bioprotectants, showing the highest rate of F5189 fungal radial growth inhibition. Therefore, these two strains were positioned as key candidates to evaluate their bioprotection efficacy under *in planta* settings. Non-bioprotectant strains were selected based on their lack of bioprotection activity against all the six *Fusarium* spp. and were chosen from the same genera as the candidate bioprotectants. We have randomly selected Lu_Sv_042 from three *Paenibacillus* strains that have demonstrated no bioprotection activity against all the six *Fusarium* spp. pathogens (Table 1). However, none of the *Pseudomonas* sp. strains screened *in vitro* had shown zero bioprotection activity against all the six isolates of *Fusarium* spp. Therefore, *Duganella* sp. strain Lu_TR935_010 was chosen, as it was the only bacterial strain from a different genus within the pool, which demonstrated zero bioprotection activity against all the six *Fusarium* spp.

### 3.2 Soil-free *in planta* pathogenicity assay

The disease rating index system was developed based on the observations made during preliminary soil-free assays and applied to both soil-free *in planta* pathogenicity and bioprotection assays (Supplementary Figure S3 and Supplementary Table S4 in Supplementary section 2). The two treatments (pathogen-challenged and negative control) across 18 lucerne cultivars showed no statistically significant difference in disease scores at 3 dpi (Supplementary Table S5). We started observing signs of stress at 6 dpi across all the cultivars when compared to their negative controls. However, statistically significantly different disease scores were recorded for F5189-pathogen challenged treatments across all 18 cultivars when compared to their negative controls on 6, 8, and 14 dpi. The highest susceptibility to Fusarium wilt was observed in Grazer cultivar at 6, 8, and 14 dpi. On the other hand, the highest resistance to Fusarium wilt was observed in Sequel cultivar at 6 and 14 dpi. However, at 8 dpi, Hunter River, Seed Force-914 and Force-5 lucerne cultivars have shown the least susceptibility to Fusarium wilt disease.

**Figure 2:**
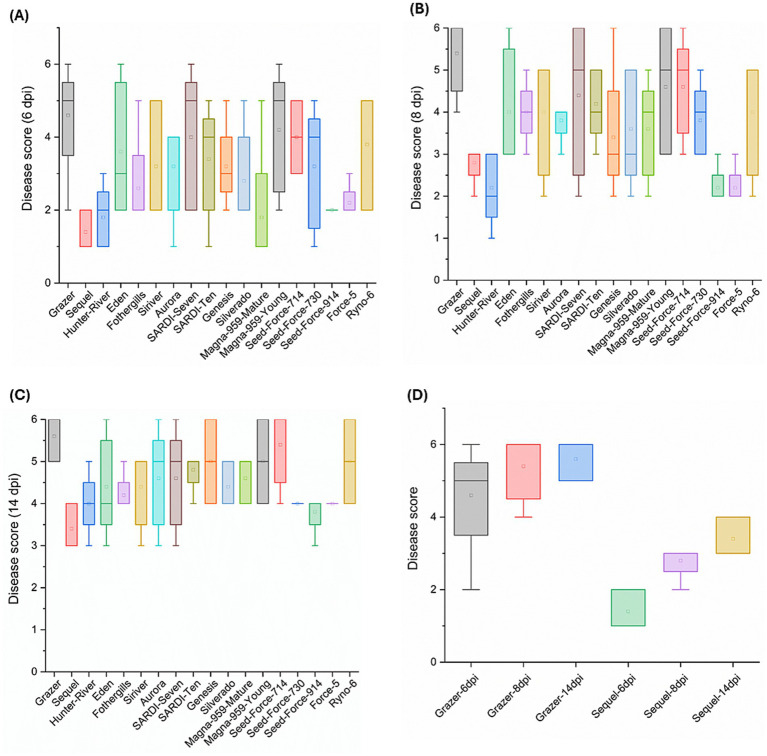
Box plots representing average disease scores and their ranges of 18 domesticated lucerne cultivars challenged with F5189 at different time points: **(A)** 6 dpi, **(B)** 8 dpi and **(C)** 14 dpi. **(D)** Comparison of average disease scores of F5189-pathogen challenged seedlings of cvs Grazer and Sequel at three different time points: 6 dpi, 8 dpi and 14 dpi. No disease symptoms were visible in controls. Hence, they are not shown in the box plot. The soil-free *in planta* pathogenicity assay consisted of five replicates each per treatment.

The disease scoring performed at the early stage of the pathogenicity assay (6 dpi) has demonstrated a high variability in the disease scores of the majority of the lucerne cultivars. For each cultivar, the disease score range is calculated as the difference between the highest and lowest scores obtained from the five replicates assessed. Based on the box plot, 13 out of 18 cultivars exhibited boxes with wide interquartile ranges resulting in a high disease score range of ≥3 (Figure 2A). By 8 dpi, the number of cultivars showing a similar disease score range has reduced to nine (Figure 2B). However, at 14 dpi, only three cultivars: Eden, Aurora and SARDI-Seven have exhibited a disease score range of ≥3 (Figure 2C).

#### 3.2.1 Selection of lucerne cultivars for *in planta* bioprotection assay

All the 18 lucerne cultivars were evaluated based on their disease scores at 14 dpi (Supplementary Table S6) to identify extremes of disease susceptibility and resistance to Fusarium wilt. Grazer exhibited the highest disease score at 14 dpi among the tested lucerne cultivars, indicating its high susceptibility to F5189 pathogen, with comparatively higher variability in its’ disease score observed at 6 dpi (Figure 2D). On the contrary, Sequel cultivar exhibited the lowest disease score, indicating high resistance to F5189 with no variability at any stage of infection. Another critical selection criterion was based on consistency and uniformity of disease response. Both Grazer and Sequel cultivars have shown consistent disease response at 14 dpi leading to more predictable and reliable performance. This uniformity has made them the most suitable candidate hosts for the *in planta* bioprotection assay.

### 3.3 Soil-free *in planta* bioprotection assay

The bioprotection efficacy of Lu_LA164_018 and Lu_MgY_007 was evaluated on both the most susceptible (cv. Grazer) and the most resistant (cv. Sequel) lucerne cultivars over a time-course which included six time points. The six treatments included in the assay were, four treatments inoculated with both bacteria and challenged with F5189 pathogen, a positive control with no bacterial inoculation and challenged with F5189 pathogen and a negative control with no inoculations of bioprotectant bacteria or pathogen.

**Figure 3:**
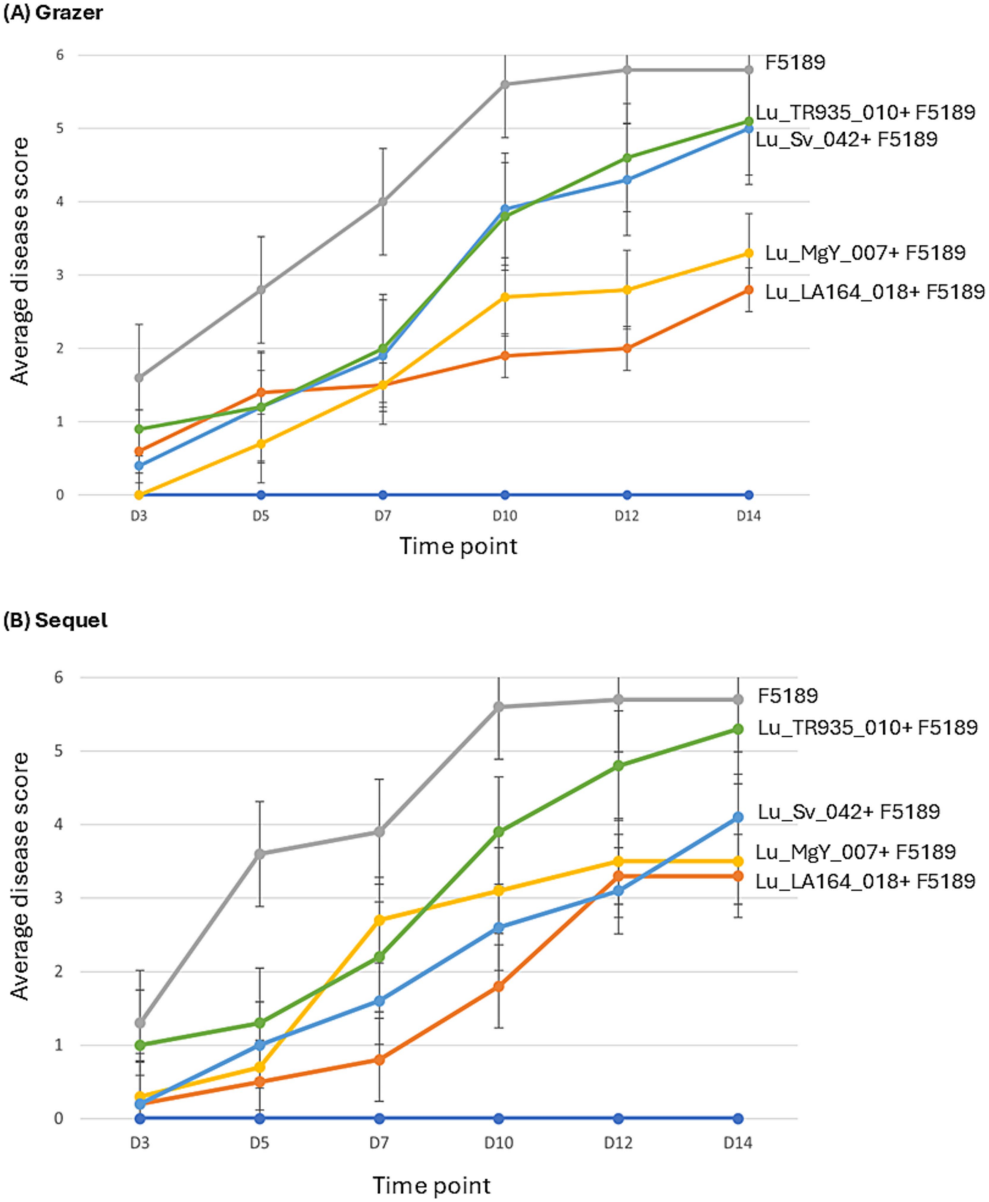
The line graphs representing the average disease scores of bioprotectant and non-bioprotectant bacterial strains and F5189 pathogen inoculated treatments, positive control inoculated only with F5189 and negative control with no bacterial and pathogen inoculations of **(A)** Grazer and **(B)** Sequel lucerne cultivars.

For Grazer, at 3 dpi Lu_MgY_007-inoculated F5189 challenged seedlings have shown a disease score of zero, while other treatments, demonstrated disease scores ranging from 0.4 to 1.6, with positive control showing the highest score (Figure 3A, Supplementary Table S7). By 5 dpi, all treatments have shown a gradual increase in disease scores except for Lu_TR935_010, which demonstrated a notable slowdown in disease progression with disease scores increased from 0.9 to 1.2. At 7 dpi, the two treatments with bioprotectants have shown completely opposite behaviours. Lu_LA164_018-inoculated F5189 challenged seedlings showed a marginal increase of 0.1 score, while Lu_MgY_007-inoculated F5189 challenged seedlings have doubled the disease score from 0.7 to 1.5. By 10 dpi, the disease scores have escalated across all the treatments except for Lu_LA164_018-inoculated F5189 challenged seedlings, which demonstrated a slight increase of disease symptoms. By 12 dpi, both bioprotectant-inoculated F5189 challenged seedlings have reached to a similar disease score, where a noticeable deceleration of disease progression was observed. In contrast, treatments with non-bioprotectants-inoculated F5189 challenged seedlings have demonstrated slight increase in their disease scores. By 14 dpi, the disease score of the positive control has reached a plateau stage, while treatments with Lu_LA164_018 and Lu_MgY_007 bioprotectant-inoculated F5189 challenged seedlings have shown modest increase in disease scores by 0.8 and 0.5, respectively. Similarly, the treatments with non-bioprotectant-inoculated F5189 challenged seedlings also demonstrated gradual increase in the disease scores.

For Sequel, the treatments with bioprotectant-inoculated F5189 challenged seedlings have exhibited low disease scores of 0.2 for Lu_LA164_018 and 0.3 for Lu_MgY_007 compared to the positive control (1.3) at 3 dpi (Figure 3B; Supplementary Table S7). By 5 dpi, the disease has escalated in the positive control (3.6), while the disease progression has noticeably slowed down in both the bioprotectant-inoculated F5189 challenged seedlings and Lu_Sv_042 non-bioprotectant-inoculated F5189 challenged seedlings. A contrast between the two bioprotectant-inoculated F5189 challenged seedlings was observed at 7 dpi, with Lu_LA164_018 slowed disease progression, while Lu_MgY_007 showed a spike in disease score from 0.7 to 2.7. A similar disease score pattern was observed for treatments with bioprotectant-inoculated F5189 challenged seedlings at 7 dpi in Grazer cultivar. At 10 dpi, the positive control and Lu_TR935_010-inoculated F5189 challenged treatment have shown a spike in the disease scores, while all other treatments exhibited a modest increase except for Lu_MgY_007-inoculated F5189 challenged seedlings, which have slowed down. At 12 dpi, the positive control exhibited a marginal increase in the disease score by 0.1, while disease progression has decelerated in Lu_MgY_007 and Lu_Sv_042-inoculated F5189 challenged treatments. By 14 dpi the disease scores of the positive control and the two treatments with bioprotectant-inoculated F5189 challenged seedlings have reached a plateau stage, while a gradual increase in disease progression was observed in non-bioprotectant-inoculated F5189 challenged treatments.

### 3.4 *In planta* pathogenicity assay in potting mix

Grazer, the most susceptible cultivar has exhibited a consistent and significant disease progression over the time-course starting from no symptoms (0) at 3 dpi to a score of 4.2 at 14 dpi (Table 2). However, there was a spike in disease score at 7 dpi from 0.9 to 2.0. Sequel has shown much lower disease scores compared to Grazer over the time-course. However, it has exhibited consistent increase in disease scores from 0 at 3 dpi to 2.7 at 14 dpi, despite being the most resistant cultivar.

**Table 2:** The average disease scores of F5189-inocultaed and negative control treatments of Grazer and Sequel lucerne cultivars at six different time points recorded in *in planta* pathogenicity assay conducted in a potting mix-based medium.

Time point	Treatment											
	Grazer_Control			Grazer_F5189+			Sequel_Control			Sequel_F5189+		
	Mean	SD	SE	Mean	SD	SE	Mean	SD	SE	Mean	SD	SE
3 dpi	0	0	0	0.0	0.00	0.00	0	0	0	0	0.00	0.00
5 dpi	0	0	0	0.9	0.32	0.10	0	0	0	0.3	0.48	0.15
7 dpi	0	0	0	2.0	0.67	0.21	0	0	0	0.4	0.52	0.16
10 dpi	0	0	0	3.1	0.88	0.28	0	0	0	1.2	0.63	0.20
12 dpi	0	0	0	3.7	0.67	0.21	0	0	0	1.8	0.79	0.25
14 dpi	0	0	0	4.2	0.42	0.13	0	0	0	2.7	0.82	0.26

### 3.5 *In planta* bioprotection assay in potting mix

**Figure 4:**
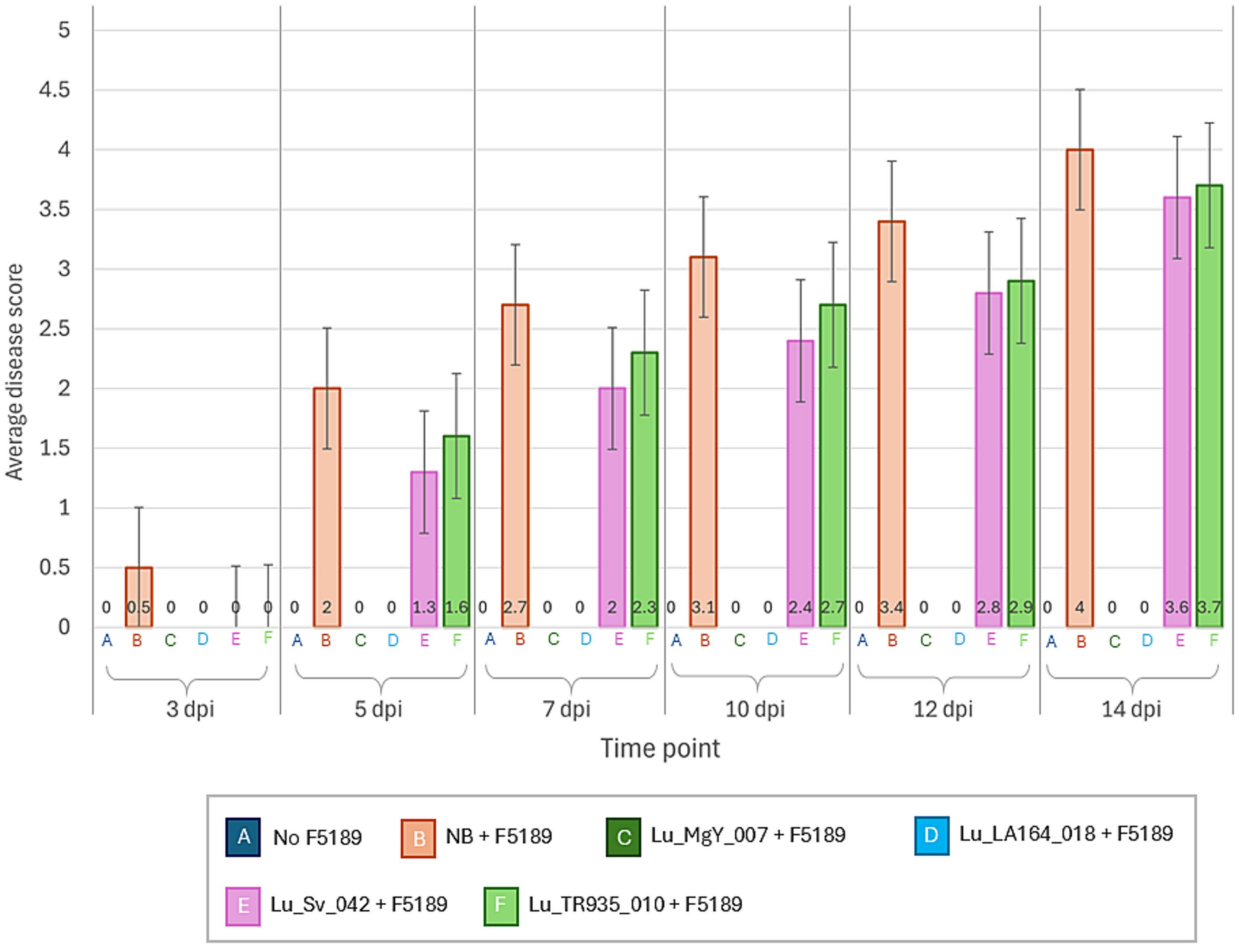
A line graph representing the average disease scores of bioprotectant and non-bioprotectant bacterial strains and F5189 pathogen inoculated treatments, positive control inoculated only with F5189 and negative control with no inoculations, in cv. Grazer in a potting mix-based medium in growth cabinets.

**Figure 5:**
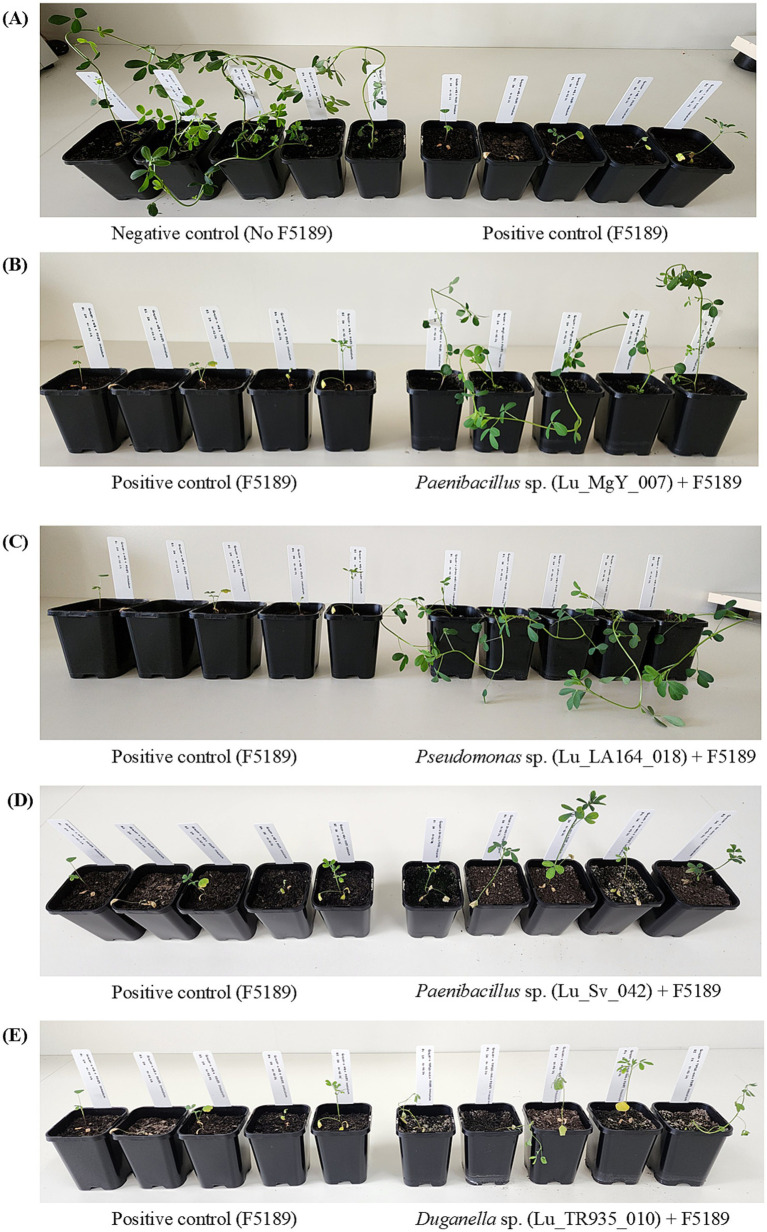
*in planta* bioprotection assay set up of cv. Grazer against F5189 pathogen conducted in growth cabinets. **(A)** Negative control with no bacterial treatment and pathogen infestation vs. positive control with only F5189 pathogen infestation. **(B)** Positive control vs. Lu_MgY_007 bioprotectant-inoculated and F5189 pathogen challenged plants. **(C)** Positive control vs. Lu_LA164_018 bioprotectant-inoculated and F5189 pathogen challenged plants. **(D)** Positive control vs. Lu_Sv_042 non-bioprotectant-inoculated and F5189 pathogen challenged plants. **(E)** Positive control vs. Lu_TR935_010 non-bioprotectants inoculated and pathogen challenged plants. The photos were taken at 14 dpi.

The experimental design implemented in the soil-free *in planta* bioprotection assay was replicated in potting mix in growth cabinets to observe the bioprotection efficacy of Lu_LA164_018 and Lu_MgY_007 on Grazer. Interestingly, none of the plants inoculated with the bioprotectants have shown disease symptoms at any time point over the time-course studied (Figures 4, 5). However, gradual progression of the disease symptoms was observed in the positive control over 14-day time-course. On the contrary, at 5 dpi, a sudden increase in disease score was observed in Lu_Sv_042 and Lu_TR935_010 non-bioprotectant-inoculated F5189 pathogen challenged treatments. In Grazer, Lu_TR935_010-inoculated F5189-pathogen challenged seedlings have shown a slowdown in disease progression at 10 dpi. However, the disease scores have increased at the same pace in the treatments with non-bioprotectants from 12 dpi to 14 dpi.

**Figure 6:**
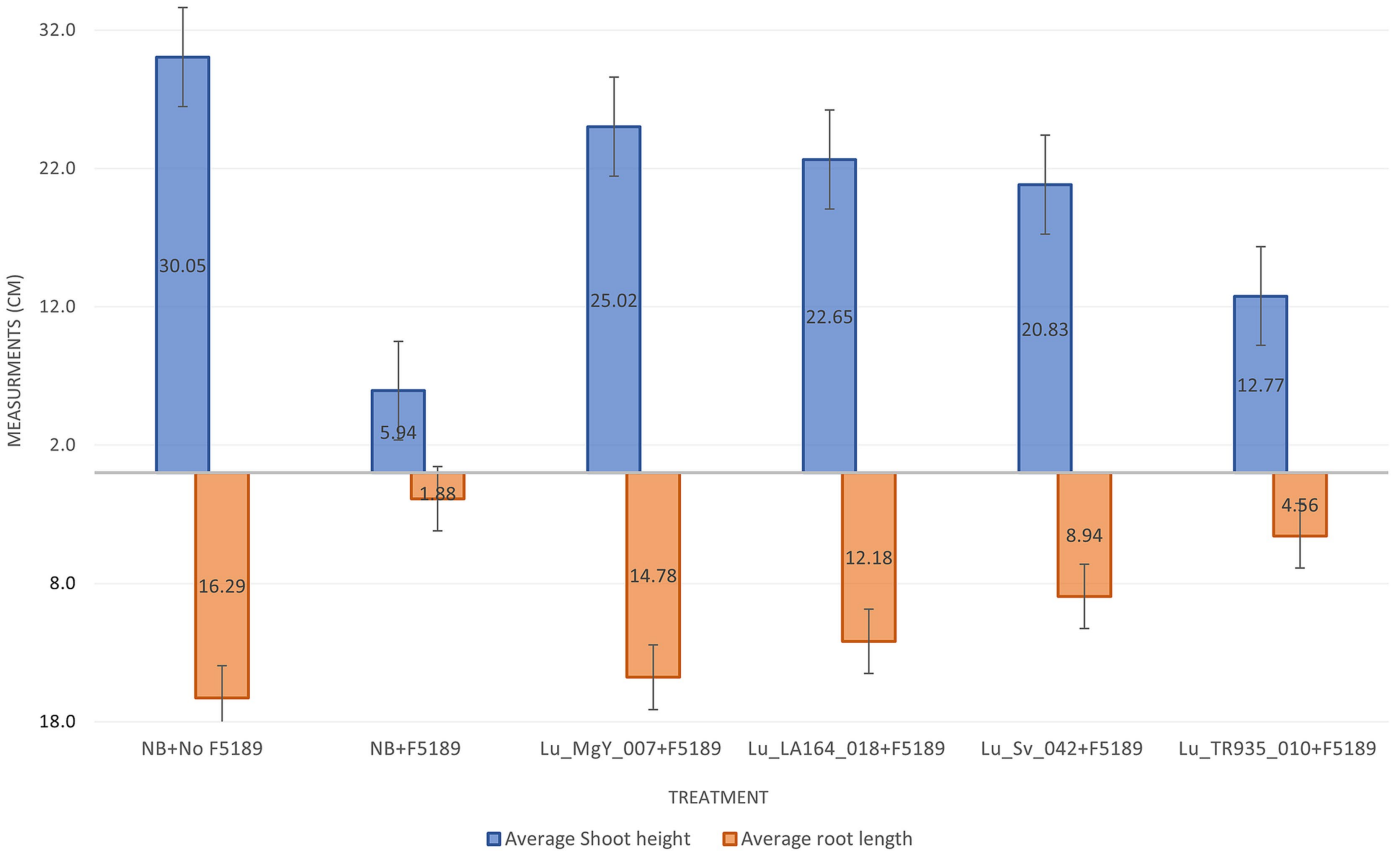
A bar graph representing average shoot and root measurements of 24 days after planting (DAP) Grazer seedlings that were assessed in potting mix-based bioprotection assay. The graph represents six inoculation systems, which included bioprotectant-treated and F5189 pathogen challenged plants, non-bioprotectant-treated and F5189 pathogen challenged plants, only F5189 pathogen challenged plants and negative control plants without bacterial inoculations and pathogen infestation.

Additionally, two growth parameters including shoot height and root length were measured in the *in planta* bioprotection assay. These measurements were taken at 14 dpi. The shoot lengths were measured from base to the youngest leaf (last leaf) and the root lengths were measured from the tip of the main root to the root collar. The average shoot height and average root lengths of F5189 pathogen challenged seedlings have decreased compared to the non-pathogen challenged seedlings by 19.77 and 11.54%, respectively, (Supplementary Table S9). However, the average shoot height of Lu_LA164_018 and Lu_MgY_007 bioprotectant-inoculated and F5189 pathogen challenged plants have increased by 3.8 and 4.8 folds, respectively, compared to the positive control (Figure 6). The corresponding average root lengths were increased by 7.9 and 6.5 folds. Similarly, the average shoot heights of Lu_Sv_042 and Lu_TR935_010 non-bioprotectant-inoculated and F5189 pathogen challenged plants have also increased by 3.5 and 2.5 folds compared to the positive control. The corresponding average root lengths were increased by 4.8 and 2.4 fold.

## 4 Discussion

Over the past decades, numerous bacterial isolates have been assessed for their bioprotection efficacy against soilborne phytopathogens. However, the lack of effective screening procedures to identify potential microbes for disease control across various soil ecosystems has resulted in the limited success of these endeavours (Pliego et al., 2011). To address this gap, our study establishes an effective screening pipeline starting from *in vitro* pre-screening to *in planta* assessment to identify promising bacterial candidates, that can mitigate Fusarium infections in commercial lucerne cultivars. Plant microbiomes act as a reservoir from which beneficial bacteria for plants can be isolated and characterized for their plant growth promoting attributes and potential bioprotection efficacy. Previous studies have demonstrated the significant potential of seed endophytes isolated from wheat (Díaz Herrera et al., 2016), rice (Cottyn et al., 2009) and Chinese liquorice (*Glycyrrhiza uralensis*; Wang et al., 2022) for their role in both biocontrol and plant growth promotion.

### 4.1 Evaluation of potential bacterial strains *in vitro* for their bioprotection efficacy

In this study, 34 bacterial strains were selected from the *Medicago* seed bacterial library based on their relative abundance and assessed for their *in vitro* bioprotection activity against *Fusarium* spp. The *in vitro* dual culture bioassay is still widely considered as one of the most affordable and efficient methods for pre-screening candidate bioprotectants, despite the labour-intensive nature of testing large bacterial libraries (Pliego et al., 2011; Shehata et al., 2016; Adeniji et al., 2019). Of the 34 isolates tested, 30 of them showed varying degrees of bioprotection against *Fusarium* pathogens, with 88.24% demonstrating significant *in vitro* biocontrol potential. This highlights the promising biocontrol capabilities of the *Medicago* seed microbiome.

Three strains belonging to *Pseudomonas* spp. have demonstrated bioprotection against all the six *Fusarium* pathogens, with inhibition rates ranging from 10.1 to 66.1%, indicating their broad-spectrum biocontrol potential. Devi et al. (2018) have reported a similar broad-spectrum biocontrol potential of *Pseudomonas aeruginosa* against *Fusarium* pathogens infecting potato (*Solanum tuberosum* L.). Similarly, several previous studies have reported effective growth inhibition of *F. oxysporum* f. sp. *ciceris* (the causal agent of chickpea wilt) by several strains of *P. fluorescens* (Inam-ul-Haq et al., 2003; Kaur et al., 2007; Kandoliya and Vakharia, 2013). The biocontrol mechanisms of *Pseudomonas* spp. include the secretion antibiotics such as pyrrolnitrinis (Thomashow and Weller, 1996; Raaijmakers et al., 2002), production of cell wall-degrading enzymes which affect the cell wall structural integrity of the target pathogen (Thangavelu et al., 2003) and biosynthesis of metabolites that induce the systemic acquired resistance (SAR) in host plants (Muthukumar et al., 2022). Interestingly, certain bacterial isolates exhibited selective bioprotection; seven out of 34 bacterial isolates inhibited the three pathogenic isolates of Fo., but showed no bioprotection against Fp. This specificity highlights the potential mechanisms and host-pathogen interactions underlying their bioprotection efficacy.

### 4.2 Pathogenicity dynamics of Fusarium wilt: cultivar susceptibility and disease progression under soil-free *in planta* settings

To the best of our knowledge, no previous study has specifically designed a pathogenicity assay for Fusarium wilt caused by Fom in lucerne plants. Hence, we believe that this pathogenicity assay addresses a significant gap in the existing literature. The main symptoms observed, including wilting and reddish-brownish discolouration of above-ground plant tissues, were consistent with those typically associated with Fusarium wilt (Nyvall, 1989), affirming the reliability of our experimental design. The assay effectively revealed variability in cultivar responses, particularly at initial infection stages (6 dpi), with 13 out of 18 cultivars demonstrating high disease score range of ≥3 (Figure 2A). This variability decreased over time, as fewer cultivars showed such high disease scores (Figures 2B,C). These findings emphasize the importance of early infection dynamics in determining overall disease outcome. Similar findings have been reported in other crop-pathogen systems, where early-stage infection was critical in disease establishment. Variability in disease scores at 6 dpi could reflect differences in initial pathogen load, pathogen dissemination rates and the timing and potency of host immune responses (Jeger et al., 2004; Xu, 2006).

Out of 18 lucerne cultivars studied, Grazer exhibited the highest susceptibility, with consistently high disease scores. This suggests delayed and insufficient host defence responses, leading to extensive pathogen colonization and symptom development (Glazebrook, 2005; Zheng et al., 2006; Thomma et al., 2011). In contrast, Sequel demonstrated the lowest scores, indicating strong resistance, which could be attributed to a rapid activation of defence mechanisms, potentially involving both innate immune responses and inducible resistance pathways (Heath, 2000; Hammond-Kosack and Parker, 2003; Jones and Dangl, 2006). While a similar study by Rispail et al. (2015), also identified cultivar-specific responses of *M. truncatula* to Fusarium wilt, their assay was tailored to *M. truncatula*, which is a CWR and is less applicable to lucerne. In contrast, our three-tiered evaluation pipeline combined with time-course analysis at multiple time points, offers deeper insights into pathogen dynamics and addresses environmental variations. Considering all this, our pipeline is more effective and directly applicable for assessing the pathogenicity of wilt pathogen in lucerne. The selection of Grazer and Sequel cultivars for the *in planta* bioprotection assays, underpins the significance of selecting the representative cultivars with extreme susceptibility and resistance to Fusarium wilt pathogen to adequately assess the efficacy of bioprotectant bacterial strains. Grazer’s high susceptibility and Sequel’s consistent resistance provide complementary backgrounds to evaluate the bioprotection efficacy. The consistent resistant traits of the host are important for assessing the bioprotectant performance (Mundt, 2014; Brown, 2015). This approach has established a reliable baseline against which the efficacy of potential bioprotectant bacteria can be measured and has reduced experimental variability, assuring that the differences in disease scores are due to the activity of bioprotectant bacteria and not to inherent genotype differences within cultivars. Accordingly, this cultivar selection strategy ensures the evaluation of bioprotectant bacteria for their effectiveness in disease suppression across various levels of disease severities. Interestingly, transient resistance was observed in cvs Hunter River, Force-5 and Seed Force-914, despite their susceptibility at other time points. This stage-specific resistance suggests the involvement of inducible defence mechanisms that are not sustained throughout the disease course (Thaler et al., 2002; Heil, 2014). This observation underscores the complexity of host-pathogen interactions, suggesting that this pattern of resistance in certain cultivars may be stage-specific and influenced by the external environmental factors (Tian et al., 2003; Boyd et al., 2013). By 14 dpi, only three cultivars (Eden, Aurora and SARDI-Seven) retained high disease scores, indicating either persistent pathogen pressure or insufficient late-stage pathogen defence responses (Heath, 2000; Dangl and Jones, 2001).

### 4.3 Evaluation of bioprotectant efficacy and cultivar response in soil-free *in planta* settings

The soil-free *in planta* bioprotection assay provided valuable insights into the differential bioprotection efficacies of the bacterial strains against F5189 in cvs. Grazer and Sequel. The bacterial strains Lu_MgY_007 and Lu_LA164_018 selected based on their strong bioprotection efficacies *in vitro*, demonstrated varying degrees of *in planta* bioprotection efficacy. In Grazer, Lu_MgY_007 exhibited an initial strong bioprotection, effectively delaying initial disease onset. However, its mid-course fluctuations, indicate the dynamic nature of bioprotection under high disease pressure. Previous studies have also demonstrated the antagonistic activity of *Paenibacillus* spp. against various phytopathogens belonging to genera *Phytophthora* (Budi et al., 2000), *Rhizoctonia*, *Alternaria* and *Fusarium* (Jung et al., 2003; Subbanna et al., 2016; El-Sayed et al., 2019) in crops such as pepper (Xu and Kim, 2016), cucumber (Zhai et al., 2021; Yang et al., 2024), strawberry (Tsai et al., 2022), tomato and wheat (Kim et al., 2020). *Paenibacillus* spp. supresses the phytopathogen growth through various mechanisms, including the production of volatile organic compounds (Xie et al., 2024), lipopeptide antibiotics such as fusaricidin (Lee et al., 2013) and polymyxin (Hsu et al., 2017), as well as hydrolytic enzymes like chitinases (Jung et al., 2003; Subbanna et al., 2016) and glucanases (Liu et al., 2016; Yang et al., 2024). Conversely, Lu_LA164_018 maintained persistent bioprotection in Grazer throughout the time-course, indicating its greater effectiveness than Lu_MgY_007. In Sequel, both Lu_MgY_007 and Lu_LA164_018 strains exhibited strong bioprotective effects at the early stages of infection. Similar to the pattern observed in Grazer, a mid-course fluctuation in bioprotection efficacy of Lu_MgY_007 was observed in Sequel. Conversely, Lu_LA164_018 showed a more consistent bioprotective effect throughout the time-course, indicating its potential for broad-spectrum bioprotection across cultivars with various genetic compositions and susceptibility levels.

It was necessary to include non-bioprotectants as negative controls to ensure comprehensive assessment of the bioprotection efficacy of bioprotectant bacterial strains (Compant et al., 2005; Chowdhury et al., 2015). The minimal bioprotection activity exhibited by Lu_Sv_042 and Lu_TR935_010 underscores the importance of rigorous control selection to validate potent bioprotectants. The bioprotection efficacies observed in the soil-free settings are consistent with the pathogen growth inhibition patterns identified during *in vitro* screening, validating the reliability of the initial screening results in predicting bioprotectant efficacy in more complex *in planta* settings.

The findings suggests that the efficacy of the bioprotectants may be influenced by the innate immune response mechanisms of the hosts. This was exemplified by the differential patterns of disease progression in both the cultivars. The initial robust bioprotection provided by Lu_MgY_007 in the Grazer might have been outperformed by the cultivar’s high susceptibility, which possibly caused the bioprotection to reduce over time. On the contrary, Sequel’s innate immune response may have synergized with Lu_LA164_018 bioprotective mechanism resulting in a prolonged and persistent disease suppression. Our findings align with the previous studies by Pieterse et al. (2014) and Berendsen et al. (2012), which indicated that the bioprotection efficacy can differ based on host genotype and the stage of disease progression. The temporal variability in bioprotection activity in some treatments may emphasize the complexity of host-pathogen interactions and the requirement for persistent microbial activity to maintain an effective long-term bioprotection (Compant et al., 2005). Moreover, the positive control (only F5189 inoculated) treatments of both Grazer and Sequel have reached a plateau phase at later stages, likely due to the controlled lab conditions, which are not ideal for continuous pathogen proliferation. Additionally, nutrient limitations experienced by both pathogen and the host in soil-free conditions could have led to the disease score stabilization. Although a slight increase in disease scores was observed in bioprotectant treatments in Grazer from 12 to 14 dpi compared to the plateaued disease scores of bioprotectant treatments in Sequel, this could be due to Grazer’s comparatively higher susceptibility to F5189. Even though the disease scores increased, they remained significantly lower than those of the positive control, indicating the efficacy of the bioprotectants.

Although the soil-free pathogenicity and bioprotection assays were conducted in similar soil-free and controlled lab conditions, a high susceptibility was observed in cv. Sequel inoculated with only F5189 pathogen (positive control) in the bioprotection assay, compared to the pathogenicity assay. This discrepancy may be attributed to the minor handling variations such as pathogen inoculum preparation, timing or plate positioning, which could influence the minor differences in pathogen growth and host responses. Potential cross-contamination or exposure to signalling molecules from adjacent bioprotectant treatments when plate handling could have induced systemic responses like priming effect (Conrath et al., 2002), altering the pathogen proliferation and host defence response. While these factors do not undermine the validity of our findings, further investigations are warranted to understand the underlying cause of these observed variations.

### 4.4 Evaluation of potting mix influence on Fusarium wilt disease progression and cultivar responses under *in planta* settings

Our findings provide significant insights into the *in planta* Fusarium wilt disease dynamics in potting mix. In the potting mix-based pathogenicity assay, the high susceptibility of Grazer to F5189 was demonstrated by the consistent and substantial progression of the disease throughout the time-course. This observation further confirmed the Grazer’s susceptibility seen in soil-free pathogenicity assay. The rapid increase in disease scores during the mid-course, further highlighted Grazer’s delayed defence response to the pathogen or suppression of host’s defence mechanism by aggressive pathogen colonization, which was also apparent in the soil-free assay. Similarly, Pieterse et al. (2014) demonstrated that the susceptible cultivars exhibited an initial delay in activating defence responses, which led to rapid symptom development as the infection progressed. Contrarily, Sequel showed lower disease scores than Grazer, which gradually increased over the time-course. This emphasizes that even highly resistant cultivars may also demonstrate partial susceptibility and are not completely immune when exposed to high pathogen pressure. The activation of defence mechanisms in resistant cultivars can compromise plant functions due to resource reallocation, which may lead to enhanced vulnerability to pathogens under stress conditions (Huot et al., 2014). Moreover, the gradual progression of the disease in Sequel implies that its defence mechanism might not completely supress pathogen proliferation. This shows the complex interactions between constitutive and inducible defence responses that pathogen could partially overcome (Dangl and Jones, 2001; Glazebrook, 2005). The results also highlight the complex nature of cultivar’s resistance. For instance, the slight increase in disease scores during the mid-course in Sequel suggests the possible influence of the potting mix on modulating pathogen virulence and host’s defence responses, which work differently than in soil-free settings (Raaijmakers et al., 2009). The soil microbial community can modulate the pathogen virulence and soil structure, and the moisture can influence the pathogen survival (Otten et al., 1999; Gill et al., 2000; Otten et al., 2004). In addition, soil physical and chemical properties such as pH (Felle et al., 2005; Kesten et al., 2019) and the nutrients such as nitrogen and potassium have a direct influence on mounting an effective host defence response against the pathogen (Amtmann et al., 2008; Mur et al., 2016; Sun et al., 2020).

### 4.5 Evaluation of bioprotectants’ efficacy and cultivar response in *in planta* bioprotection assay in potting mix

The outcomes of *in planta* bioprotection assay in potting mix indicate that the potential bioprotectant strains; Lu_LA164_018 and Lu_MgY_007 have effectively mitigated the Fusarium wilt disease symptoms in cv. Grazer across all time points under potting mix conditions. This contrasted with the progressive disease symptoms observed in the positive control. These findings are consistent with previous studies by Backer et al. (2018) demonstrating similar bioprotective effects in soil-based assays. On the other hand, the variability in bioprotection efficacy observed in soil-free conditions can be attributed to controlled environmental conditions under the laboratory settings, which might have influenced the effectiveness of the bioprotectants. Overall, high disease scores of Lu_Sv_042 and Lu_TR935_010 (non-bioprotectants) and specifically increased disease severity at 5 dpi highlight the effectiveness of the bioprotectant inoculated treatments. However, slow down of disease progression of Lu_TR935_010 inoculated treatment at 10 dpi suggests that potting mix might influence certain bioprotectant’s effectiveness.

Growth measurements further emphasize the significant effects of bioprotectants on improving shoot and root growth under pathogen stress compared to the non-bioprotectant treatments. The previous studies have also found that the bioprotectants can enhance the growth of pathogen stressed plants (Odoh, 2017). The variations observed between the soil-free conditions and potting mix conditions highlights the influence of plant growth conditions on the performance of bioprotectants.

In conclusion, our study revealed that the seed-associated bacteria of *Medicago* spp. are a promising avenue for discovering bioprotectants against Fusarium wilt of lucerne. Their natural adaptation to combat *Fusarium* infections, qualify them as promising biological alternatives to traditional chemical fungicides. Our three-tiered evaluation pipeline revealed noticeable variations in biocontrol efficacies depending on the plant growth medium and host-pathogen dynamics. Both bacterial candidates; *Paenibacillus* sp. (Lu_MgY_007) and *Pseudomonas* sp. (Lu_LA164_018) evaluated through the pipeline have shown significant growth inhibition of F5189 pathogen across all the assay systems studied. The soil-free assays provided preliminary understanding of the host-pathogen and bioprotectant interactions in an *in planta* setting. Although this assay has some limitations, it provided valuable insights. We have developed a potting mix-based assay by addressing all these constraints of the soil-free system, which offered a more realistic evaluation of the bioprotection efficacy. However, under soil-free conditions, these strains effectively reduced disease symptoms in lucerne cultivars irrespective of its susceptibility, while in potting mix, they completely prevented disease over the course of the assay, highlighting their strong biocontrol potential. This could be possibly due to synergistic interactions with the potting mix microbiota, enhancing their efficacy (Ptaszek et al., 2023). The temporal progression of bioprotection revealed by the time-course studies emphasis the requirement of persistent influence of bioprotectants to obtain long-term disease suppression. Our findings indicate the importance of considering the plant growth conditions, biological complexity and temporal aspects of host-pathogen interactions, when developing and introducing new microbial-based fungicidal alternates for crop disease management. This study lays the groundwork for future field trials to validate these results in real-world agricultural settings, where long-term efficacy of these bioprotectants can be further evaluated. Future studies should focus on molecular level analyses to elucidate the mechanism underpinning bioprotectant efficacy, including their roles in modulating host immune responses and suppressing pathogen virulence. Time-course transcriptomic and metabolomic studies could provide critical insights into the temporal dynamics of host-pathogen-bioprotectant interactions. This understanding would be useful in developing more precise, time-sensitive and durable biocontrol strategies to enhance sustainable agricultural practices. Additionally, optimizing bioprotectant formulation and delivery methods for scalable application in agricultural practices will be essential for their successful integration into crop disease management strategies.

## Data Availability

The datasets presented in this study can be found in online repositories. The names of the repository/repositories and accession number(s) can be found in the article/Supplementary material.
