# Supplementary material for: Identification of in planta bioprotectants against Fusarium wilt in Medicago sativa L. (lucerne) from a collection of bacterial isolates derived from Medicago seeds
Source: Front Microbiol. 2025 Feb 26;16:1544521. doi: 10.3389/fmicb.2025.1544521 (PMC11897269; doi:10.3389/fmicb.2025.1544521)
Supplement: Supplementary file 1 [file Data_Sheet_1.docx]

**Identification of *in planta* bioprotectants against Fusarium wilt in *Medicago sativa* L. (lucerne) from a collection of bacterial isolates derived from *Medicago* seeds**

Supplementary Material

# Supplementary Data file 1

Supplementary Table S1. Commercial *Medicago* (lucerne) and *Medicago* crop wild relative seed accessions used in the study. Bacterial isolations were performed on the seed accessions marked with *

|  |  | *Medicago* seed accession/cultivar | Description | Accession code | Origin | Cumulative percentage germination |
| --- | --- | --- | --- | --- | --- | --- |
| 1 | Commercial *Medicago* cultivars | *Green Harvest_Sequel** | *Medicago sativa* | Sq | Australia | 100.0% |
| 2 |  | *Green Harvest_Hunter River** | *Medicago sativa* | HR | Australia | 98.6% |
| 3 |  | *Eden Seeds_ Trifecta** | *Medicago sativa* | Ed | Australia | 75.0% |
| 4 |  | *Australian Wheatgrass_ Aurora** | *Medicago sativa* | Au | Australia | 100.0% |
| 5 |  | *Healthforce_ Siriver** | *Medicago sativa* | Sv | Australia | 95.0% |
| 6 |  | *AGF Seeds_ Ryno 6** | *Medicago sativa* | R6 | Australia | 94.3% |
| 7 |  | *AGF Seeds_ Force 5** | *Medicago sativa* | F5 | Australia | 93.6% |
| 8 |  | *Barenbrug_ SARDI SEVENv2* | *Medicago sativa* | SS | Australia | 92.1% |
| 9 |  | *Barenbrug_ SARDI TENv2* | *Medicago sativa* | ST | Australia | 94.7% |
| 10 |  | *Barenbrug_ SARDI GRAZER* | *Medicago sativa* | SG | Australia | 92.6% |
| 11 |  | *Barenbrug_ GENESIS* | *Medicago sativa* | GN | Australia | 93.8% |
| 12 |  | *Upper Murray Seeds _ Silverado* | *Medicago sativa* | SL | Australia | 95.2% |
| 13 |  | *Mr Fothergills_ Sprouts Alive** | *Medicago sativa* | Ft | Australia | 58.6% |
| 14 |  | *Magna 959 (mature seeds)* | *Medicago sativa* | MM | Australia | 22.0% |
| 15 |  | *Magna 959 (young seeds)** | *Medicago sativa* | MY | Australia | 15.0% |
| 16 |  | *Seed Force_ Seed Force 714* | *Medicago sativa* | SF714 | Australia | 80.3% |
| 17 |  | *Seed Force_ Seed Force 730* | *Medicago sativa* | SF730 | Australia | 82.4% |
| 18 |  | *Seed Force_ Seed Force 914* | *Medicago sativa* | SF914 | Australia | 80.9% |
| 19 | *Medicago* CWR cultivars | *APG 6032* | *Medicago sativa subsp. falcata* | FL32 | Russia | 90.2% |
| 20 |  | *APG 6039* | *Medicago sativa subsp. falcata* | FL39 | Russia | 92.1% |
| 21 |  | *APG 6925* | *Medicago sativa subsp. falcata* | FL25 | Russia | 90.9% |
| 22 |  | *APG 20535* | *Medicago littoralis var. littoralis* | LTV_535 | Libya | 94.7% |
| 23 |  | *APG 21384* | *Medicago littoralis var. littoralis* | LTV_384 | Libya | 95.0% |
| 24 |  | *APG 21559* | *Medicago littoralis var. littoralis* | LTV_559 | Libya | 94.2% |
| 25 |  | *APG 32892* | *Medicago littoralis var. littoralis* | LTV_892 | Libya | 95.9% |
| 26 |  | *APG 21164** | *Medicago laciniata* | LA164 | Libya | 96.0% |
| 27 |  | *APG 21177* | *Medicago laciniata* | LA177 | Libya | 96.8% |
| 28 |  | *APG 20841** | *Medicago laciniata* | LA841 | Libya | 94.9% |
| 29 |  | *APG 21700** | *Medicago laciniata* | LA700 | Libya | 95.6% |
| 30 |  | *APG 20935** | *Medicago truncatula* | TR935 | Libya | 93.5% |
| 31 |  | *APG 21758** | *Medicago truncatula* | TR758 | Libya | 94.2% |
| 32 |  | *APG 21771** | *Medicago truncatula* | TR771 | Libya | 94.9% |
| 33 |  | *APG 21177** | *Medicago littoralis* | LT177 | Libya | 82.3% |
| 34 |  | *APG 21198** | *Medicago littoralis* | LT198 | Libya | 74.1% |
| 35 |  | *APG 21232** | *Medicago littoralis* | LT232 | Libya | 78.3% |
| 36 |  | *APG 21235** | *Medicago littoralis* | LT235 | Libya | 75.3% |

**Supplementary Table S2.** The closest 16S rRNA gene sequence match (NCBI BLAST) of candidate bacterial isolates recovered from *Medicago* seeds. The full-length 16S sequences of the 34 bacterial strains were deposited in the NCBI GenBank under the BioProject PRJNA1180717.

| Bacterial isolate ID | Host plant species | The closest 16S rRNA gene sequence match (NCBI BLAST) | Percentage identity | Updated GenBank accession numbers of the isolates |
| --- | --- | --- | --- | --- |
| Lu_Au_053 | *M. sativa* | *Enterobacter kobei* | 99.64% | PQ756878 |
| Lu_Au_058 | *M. sativa* | *Enterobacter* sp. MLB27 | 99.92% | PQ756879 |
| Lu_F5_006 | *M. sativa* | *Pseudomonas koreensis* | 100.00% | PQ756880 |
| Lu_F5_008 | *M. sativa* | *Massilia* sp. 4D10 | 99.78% | PQ756881 |
| Lu_F5_028 | *M. sativa* | *Duffyella gerundensis* | 99.93% | PQ756882 |
| Lu_F5_029 | *M. sativa* | *Pseudomonas* sp. XBBSY4 | 99.93% | PQ756883 |
| Lu_MgY_007 | *M. sativa* | *Paenibacillus terrae* | 99.72% | PQ756884 |
| Lu_R6_023 | *M. sativa* | *Duffyella gerundensis* | 99.86% | PQ756885 |
| Lu_Sv_042 | *M. sativa* | *Paenibacillus* sp. FSL E2-0151 | 99.71% | PQ756886 |
| Lu_LA164_018 | *M. laciniata* | *Pseudomonas* sp. R11-45-07 | 97.40% | PQ756887 |
| Lu_LA164_009 | *M. laciniata* | *Duganella* sp. PH3 | 97.59% | PQ756888 |
| Lu_LA164_012 | *M. laciniata* | *Duganella* sp. PH3 | 96.59% | PQ756889 |
| Lu_LA164_003 | *M. laciniata* | *Pantoea agglomerans* | 98.70% | PQ756890 |
| Lu_LA700_W009 | *M. laciniata* | *Pantoea sp.* | 98.82% | PQ756891 |
| Lu_LA841_007 | *M. laciniata* | Enterobacteriaceae bacterium SAP758.2 | 98.84% | PQ756892 |
| Lu_LA841_009 | *M. laciniata* | Enterobacteriaceae bacterium SAP758.2 | 98.35% | PQ756893 |
| Lu_LA841_015 | *M. laciniata* | *Pseudomonas* sp. Na1 | 97.25% | PQ756894 |
| Lu_LT177_010 | *M. littoralis* | *Paenibacillus xylanexedens* | 99.49% | PQ756895 |
| Lu_LT198_002 | *M. littoralis* | Enterobacteriaceae bacterium SAP758.2 | 99.71% | PQ756896 |
| Lu_LT198_003 | *M. littoralis* | *Pantoea agglomerans* | 99.90% | PQ756897 |
| Lu_LT198_010 | *M. littoralis* | *Pseudomonas ovata* | 99.42% | PQ756898 |
| Lu_LT198_018 | *M. littoralis* | Enterobacteriaceae bacterium SAP758.2 | 99.71% | PQ756899 |
| Lu_LT198_042 | *M. littoralis* | *Paenibacillus nicotianae* | 99.29% | PQ756900 |
| Lu_LT198_W003 | *M. littoralis* | *Pseudomonas lutea* | 99.86% | PQ756901 |
| Lu_LT235_004 | *M. littoralis* | *Pantoea agglomerans* | 99.43% | PQ756902 |
| Lu_TR758_007 | *M. truncatula* | Enterobacteriaceae bacterium SAP758.2 | 99.42% | PQ756903 |
| Lu_TR758_008 | *M. truncatula* | *Paenibacillus* sp. 3Cp1 | 100.00% | PQ756904 |
| Lu_TR758_011 | *M. truncatula* | *Pseudomonas* sp. SAP829.3 | 99.12% | PQ756905 |
| Lu_TR758_015 | *M. truncatula* | Enterobacteriaceae bacterium SAP758.2 | 99.64% | PQ756906 |
| Lu_TR758_W005 | *M. truncatula* | *Pantoea eucalypti* | 98.58% | PQ756907 |
| Lu_TR771_006 | *M. truncatula* | *Pantoea agglomerans* | 99.29% | PQ756908 |
| Lu_TR771_007 | *M. truncatula* | *Paenibacillus* sp. 3Cp1 | 97.63% | PQ756909 |
| Lu_TR935_010 | *M. truncatula* | *Duganella* sp. PH3 | 99.78% | PQ756910 |
| Lu_TR935_014 | *M. truncatula* | *Pseudomonas* sp. | 96.96% | PQ756911 |

* A total of 34 bacterial isolates were chosen based on a bacterial species-*Medicago* host matrix to examine the potential biological control attributes. These isolates were identified and characterized by 16S rRNA sequences obtained from Sanger sequencing. The criteria for selection were members of bacterial genera that were present in more than one *Medicago* host species based on 16S *Medicago* seed microbiome profiling data (Herath D. et al., 2025, Manuscript in preparation). Those selected genera included isolates belonging to *Pantoea*, *Paenibacillus*, *Duffyella*, *Pseudomonas*, *Massilia*, *Duganella* and family Enterobacteriaceae. However, the genus, *Sphingomonas* was excluded from the list due to the absence of isolates belonging to *Sphingomonas* in our *Medicago* bacterial library.

Supplementary Table S3. *Fusarium* pathogenic strains used in the study

| VPRI Accession | Taxonomic Details | Host Taxonomic Details | State | Date of Collection |
| --- | --- | --- | --- | --- |
| 42191 | *Fusarium proliferatum* | *Solanum lycopersicum* L. | VIC | 15/01/2013 |
| 42409 | *Fusarium proliferatum* | *Asparagus officinalis* | VIC | 30/06/2014 |
| 42958 | *Fusarium proliferatum* | NA | VIC | NA |
| 44256 (5190) | *Fusarium oxysporum* f. sp. *medicaginis* | *Medicago sativa* | QLD | NA |
| 44314 | *Fusarium oxysporum* | *Medicago sativa* | SA | 7/06/2020 |
| 44257 (5189) | *Fusarium oxysporum* f. sp. *medicaginis* | *Medicago sativa* | QLD | Dec-72 |

Supplementary Table S5. The *Fusarium* wilt disease scores of F5189-pathogen challenged treatments and negative control treatments of 18 lucerne cultivars at four different time points in soil-free *in planta* pathogenicity assay

| **Treatment** | **Time point** | | | | | | | |
| --- | --- | --- | --- | --- | --- | --- | --- | --- |
|  | 3 dpi | | 6 dpi | | 8 dpi | | 14 dpi | |
|  | Mean | SE of Mean | Mean | SE of Mean | Mean | SE of Mean | Mean | SE of Mean |
| Hunter-River-Control | 0 | 0 | 0 | 0.00 | 0 | 0.00 | 0 | 0.00 |
| Hunter-River-F5189+ | 0 | 0 | 1.8 | 0.37 | 2.2 | 0.37 | 4 | 0.32 |
| Sequel-Control | 0 | 0 | 0 | 0.00 | 0 | 0.00 | 0 | 0.00 |
| Sequel-F5189+ | 0 | 0 | 1.4 | 0.24 | 2.8 | 0.20 | 3.4 | 0.24 |
| Eden-Control | 0 | 0 | 0 | 0.00 | 0 | 0.00 | 0 | 0.00 |
| Eden-F5189+^c^ | 0 | 0 | 3.6 | 0.81 | 4 | 0.63 | 4.4 | 0.51 |
| Fothergills-Control | 0 | 0 | 0 | 0.00 | 0 | 0.00 | 0 | 0.00 |
| Fothergills-F5189+ | 0 | 0 | 2.6 | 0.60 | 4 | 0.32 | 4.2 | 0.20 |
| Siriver-Control | 0 | 0 | 0 | 0.00 | 0 | 0.00 | 0 | 0.00 |
| Siriver-F5189+ | 0 | 0 | 3.2 | 0.73 | 4 | 0.63 | 4.4 | 0.40 |
| Aurora-Control | 0 | 0 | 0 | 0.00 | 0 | 0.00 | 0 | 0.00 |
| Aurora-F5189+ | 0 | 0 | 3.2 | 0.58 | 3.8 | 0.20 | 4.6 | 0.51 |
| SARDI-Seven-Control | 0 | 0 | 0 | 0.00 | 0 | 0.00 | 0 | 0.00 |
| SARDI-Seven-F5189+ | 0 | 0 | 4 | 0.84 | 4.4 | 0.81 | 4.6 | 0.51 |
| SARDI-Ten-Control | 0 | 0 | 0 | 0.00 | 0 | 0.00 | 0 | 0.00 |
| SARDI-Ten-F5189+ | 0 | 0 | 3.4 | 0.68 | 4.2 | 0.37 | 4.8 | 0.20 |
| Genesis-Control | 0 | 0 | 0 | 0.00 | 0 | 0.00 | 0 | 0.00 |
| Genesis-F5189+ | 0 | 0 | 3.2 | 0.49 | 3.4 | 0.68 | 5 | 0.45 |
| Silverado-Control | 0 | 0 | 0 | 0.00 | 0 | 0.00 | 0 | 0.00 |
| Silverado-F5189+ | 0 | 0 | 2.8 | 0.58 | 3.6 | 0.60 | 4.4 | 0.24 |
| Magna-959-Mature-Control | 0 | 0 | 0 | 0.00 | 0 | 0.00 | 0 | 0.00 |
| Magna-959-Mature-F5189+ | 0 | 0 | 1.8 | 0.80 | 3.6 | 0.51 | 4.6 | 0.24 |
| Magna-959-Young-Control | 0 | 0 | 0 | 0.00 | 0 | 0.00 | 0 | 0.00 |
| Magna-959-Young-F5189+ | 0 | 0 | 4.2 | 0.73 | 4.6 | 0.68 | 5 | 0.45 |
| Seed-Force-714-Control | 0 | 0 | 0 | 0.00 | 0 | 0.00 | 0 | 0.00 |
| Seed-Force-714-F5189+ | 0 | 0 | 4 | 0.45 | 4.6 | 0.51 | 5.4 | 0.40 |
| Seed-Force-730-Control | 0 | 0 | 0 | 0.00 | 0 | 0.00 | 0 | 0.00 |
| Seed-Force-730-F5189+ | 0 | 0 | 3.2 | 0.73 | 3.8 | 0.37 | 4 | 0.00 |
| Seed-Force-914-Control | 0 | 0 | 0 | 0.00 | 0 | 0.00 | 0 | 0.00 |
| Seed-Force-914-F5189+ | 0 | 0 | 2 | 0.00 | 2.2 | 0.20 | 3.8 | 0.20 |
| Force-5-Control | 0 | 0 | 0 | 0.00 | 0 | 0.00 | 0 | 0.00 |
| Force-5-F5189+ | 0 | 0 | 2.2 | 0.20 | 2.2 | 0.20 | 4 | 0.00 |
| Ryno-6-Control | 0 | 0 | 0 | 0.00 | 0 | 0.00 | 0 | 0.00 |
| Ryno-6-F5189+ | 0 | 0 | 3.8 | 0.73 | 4 | 0.63 | 5 | 0.45 |
| Grazer-Control | 0 | 0 | 0 | 0.00 | 0 | 0.00 | 0 | 0.00 |
| Grazer-F5189+ | 0 | 0 | 4.6 | 0.68 | 5.4 | 0.40 | 5.6 | 0.24 |

Supplementary Table S6. The *Fusarium* wilt disease scores of F5189-inoculated and negative control treatments of 18 lucerne cultivars at 14 dpi, ranked from highest to lowest susceptibility (Soil-free *in planta* bioprotection assay)

| **Treatment** | **Mean** | **Standard Deviation** | **SE of Mean** |
| --- | --- | --- | --- |
| Grazer-F5189+ | 5.6 | 0.55 | 0.24 |
| Seed-Force-714-F5189+ | 5.4 | 0.89 | 0.40 |
| Genesis-F5189+ | 5 | 1.00 | 0.45 |
| Magna-959-Young-F5189+ | 5 | 1.00 | 0.45 |
| Ryno-6-F5189+ | 5 | 1.00 | 0.45 |
| SARDI-Ten-F5189+ | 4.8 | 0.45 | 0.20 |
| Aurora-F5189+ | 4.6 | 1.14 | 0.51 |
| SARDI-Seven-F5189+ | 4.6 | 1.14 | 0.51 |
| Magna-959-Mature-F5189+ | 4.6 | 0.55 | 0.24 |
| Eden-F5189+ | 4.4 | 1.14 | 0.51 |
| Siriver-F5189+ | 4.4 | 0.89 | 0.40 |
| Silverado-F5189+ | 4.4 | 0.55 | 0.24 |
| Fothergills-F5189+ | 4.2 | 0.45 | 0.20 |
| Hunter-River-F5189+ | 4 | 0.71 | 0.32 |
| Seed-Force-730-F5189+ | 4 | 0.00 | 0.00 |
| Force-5-F5189+ | 4 | 0.00 | 0.00 |
| Seed-Force-914-F5189+ | 3.8 | 0.45 | 0.20 |
| Sequel-F5189+ | 3.4 | 0.55 | 0.24 |
| Hunter-River-Control | 0 | 0.00 | 0.00 |
| Sequal-Control | 0 | 0.00 | 0.00 |
| Eden-Control | 0 | 0.00 | 0.00 |
| Fothergills-Control | 0 | 0.00 | 0.00 |
| Siriver-Control | 0 | 0.00 | 0.00 |
| Aurora-Control | 0 | 0.00 | 0.00 |
| SARDI-Seven-Control | 0 | 0.00 | 0.00 |
| SARDI-Ten-Control | 0 | 0.00 | 0.00 |
| Genesis-Control | 0 | 0.00 | 0.00 |
| Silverado-Control | 0 | 0.00 | 0.00 |
| Magna-959-Mature-Control | 0 | 0.00 | 0.00 |
| Magna-959-Young-Control | 0 | 0.00 | 0.00 |
| Seed-Force-714-Control | 0 | 0.00 | 0.00 |
| Seed-Force-730-Control | 0 | 0.00 | 0.00 |
| Seed-Force-914-Control | 0 | 0.00 | 0.00 |
| Force-5-Control | 0 | 0.00 | 0.00 |
| Ryno-6-Control | 0 | 0.00 | 0.00 |
| Grazer-Control | 0 | 0.00 | 0.00 |

Supplementary Table S9. Average shoot and root measurements of 24 DAP Grazer (Gr) seedlings of 14 dpi challenged with F5189 pathogen. The table represents six inoculation systems, which included two of the bioprotectant-inoculated and F5189 pathogen challenged treatments, two of the non-bioprotectant-inoculated and F5189 pathogen challenged treatments, only F5189 pathogen challenged treatment and negative control treatment (NC) without bacterial treatments and pathogen infestation.

| **Treatment** | **Average shoot/root measurement (cm)** | | |
| --- | --- | --- | --- |
|  | Mean | Standard deviation | SE of mean |
| Shoot | | | |
| Gr-NC | 30.05 | 6.04 | 1.91 |
| Gr-F5189 | 5.94 | 4.17 | 1.32 |
| Gr-Lu_LA164_018-F5189 | 22.65 | 2.50 | 0.79 |
| Gr-Lu_MgY_007-F5189 | 25.02 | 2.58 | 0.82 |
| Gr-Lu_Sv_042-F5189 | 20.83 | 2.51 | 0.79 |
| Gr-Lu_TR935_010-F5189 | 12.77 | 2.89 | 0.91 |
| Root | | | |
| Gr-NC | 16.29 | 2.18 | 0.69 |
| Gr-F5189 | 1.88 | 1.13 | 0.36 |
| Gr-Lu_LA164_018-F5189 | 12.18 | 1.98 | 0.63 |
| Gr-Lu_MgY_007-F5189 | 14.78 | 1.93 | 0.61 |
| Gr-Lu_Sv_042-F5189 | 8.94 | 1.57 | 0.50 |
| Gr-Lu_TR935_010-F5189 | 4.56 | 1.04 | 0.33 |

| Bacterial isolate ID |  | Rep 1 |  | Rep 2 |  | Rep 3 |
| --- | --- | --- | --- | --- | --- | --- |
| Control |  | 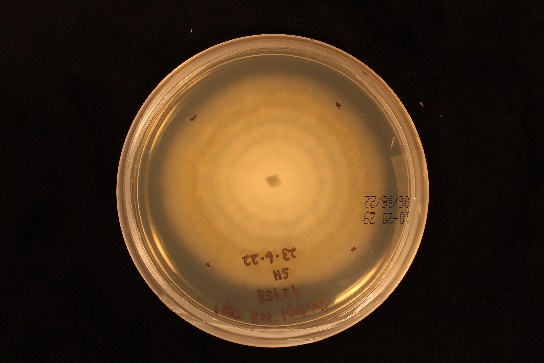 |  | 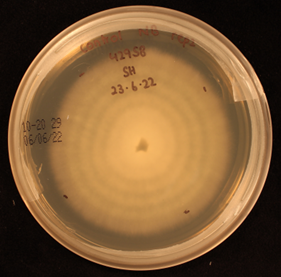 |  | 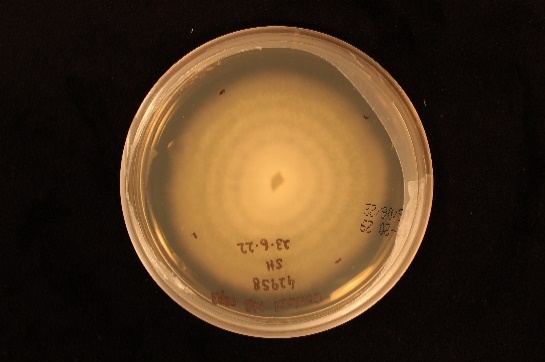 |
| Lu_MgY_007 |  | 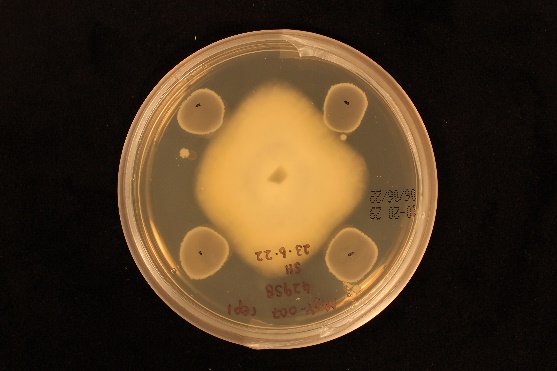 |  | 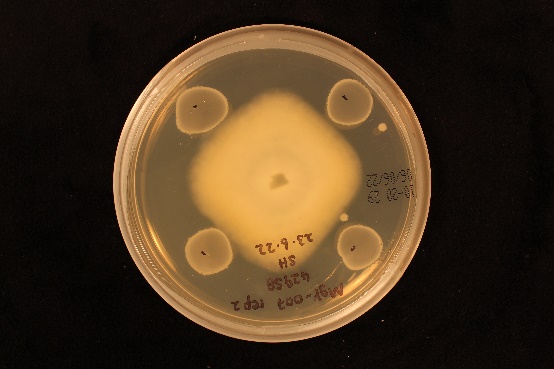 |  | 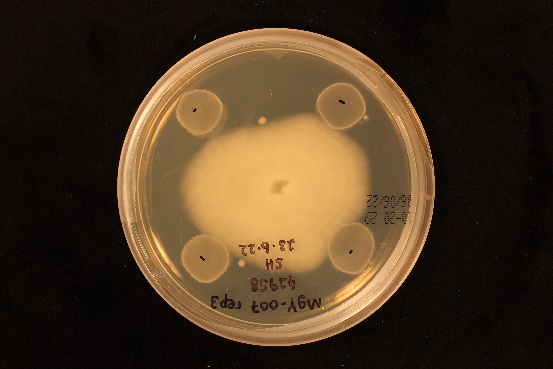 |
| Lu_TR758_007 |  | 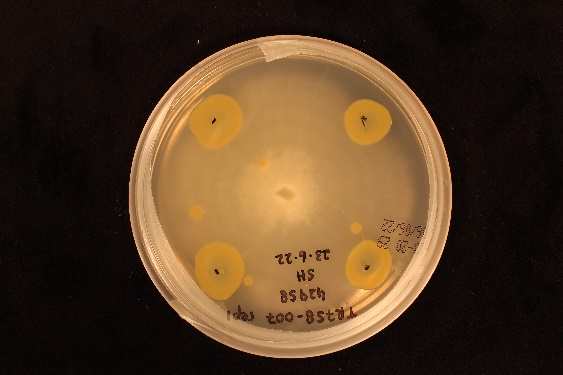 |  | 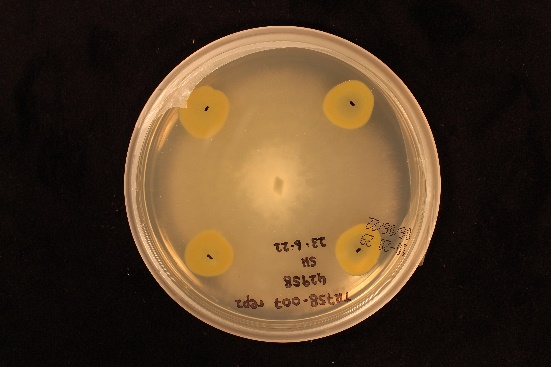 |  | 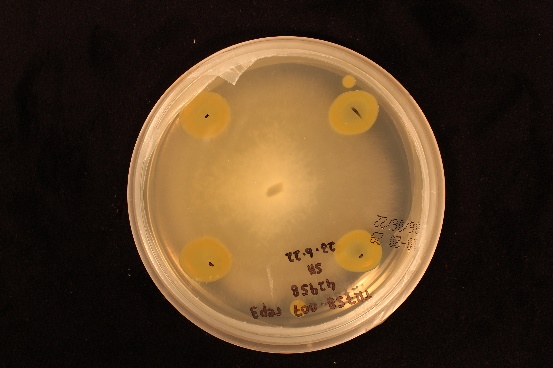 |

Supplementary Figure S1. Representative images of the *in vitro* bioprotection assay of bacterial

strains Lu_MgY_007 and Lu_TR758_007 against *F. proliferatum* 42958 phytopathogen

Supplementary Figure S2. Representative images of the *in vitro* bioprotection assay of bacterial strains Lu_LA164_018 and Lu_LA164_009 against *F. oxysporum* 44256 (5190) phytopathogen

| Bacterial isolate ID |  | Rep 1 |  | Rep 2 |  | Rep 3 |
| --- | --- | --- | --- | --- | --- | --- |
| Control |  | 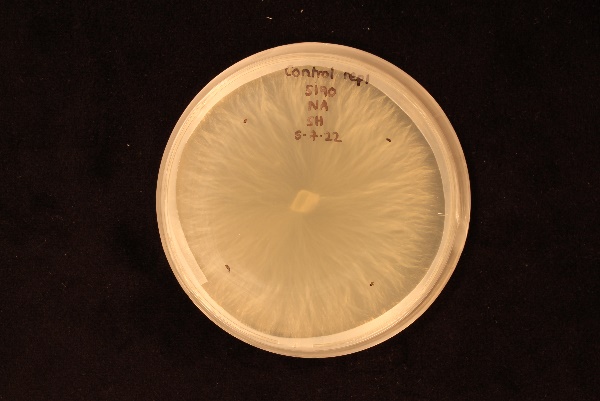 |  | 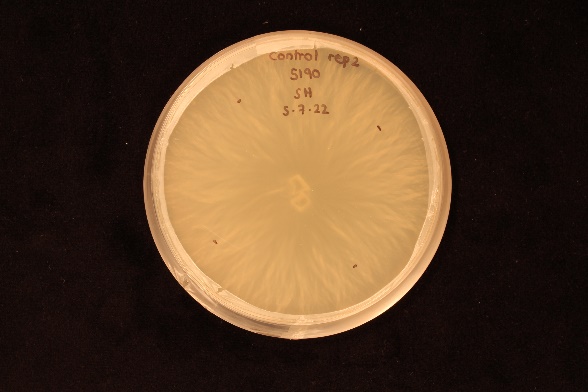 |  | 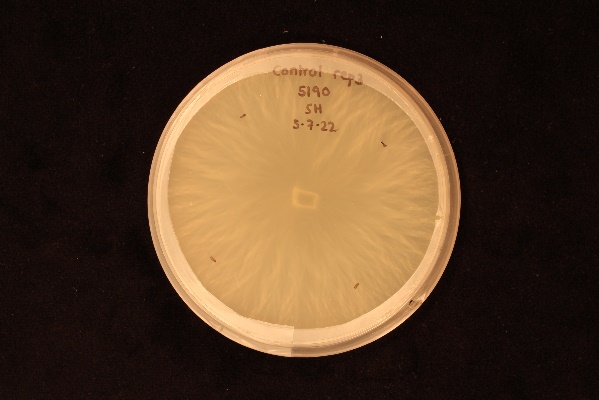 |
| Lu-LA164-018 |  | 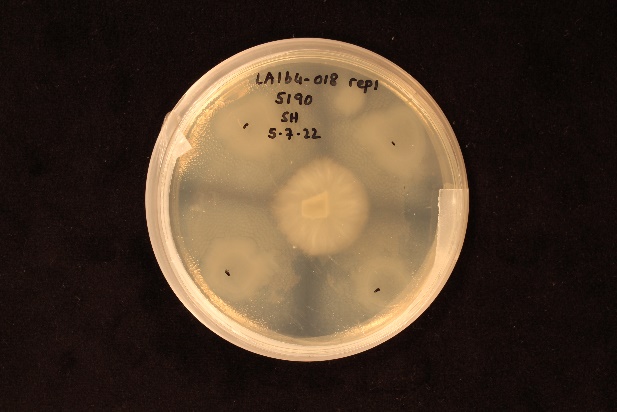 |  | 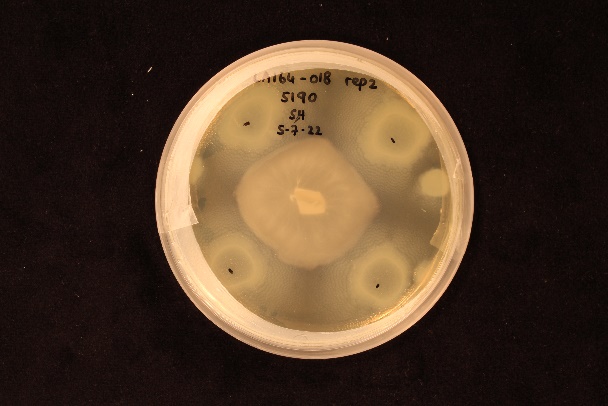 |  | 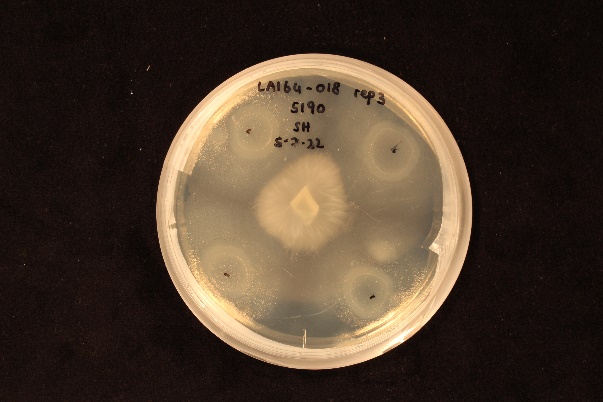 |
| Lu_LA164_009 |  | 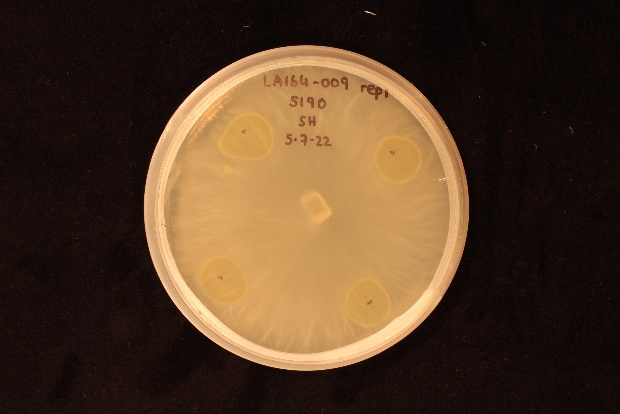 |  | 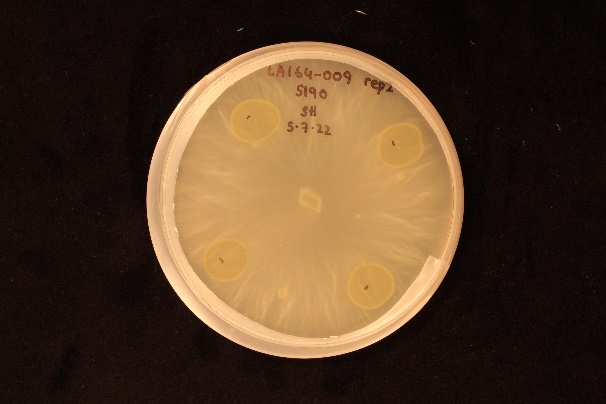 |  | 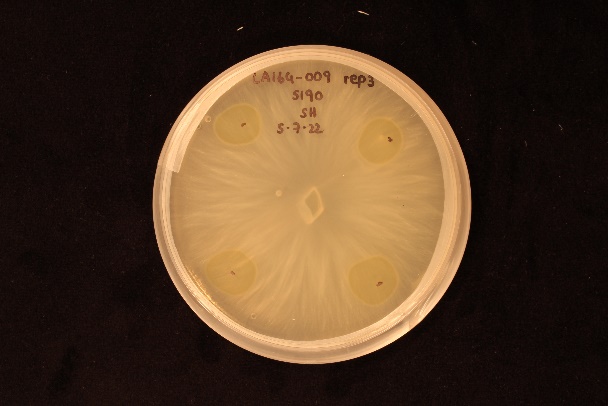 |

**Supplementary section 1**

*Fusarium* inoculum preparation

The F5189 fungal inoculum was prepared by using sterile millet seeds. For sterilization, approximately 450g of millet grains were placed in a Schott bottle and washed three times with sterile distilled water. The washed millet grains were soaked in sterile distilled water overnight. After overnight soaking, millet grains were transferred into a new Schott bottle and autoclaved for 1 hour at 121°C twice with cooling down in between each sterilization. F5189 was grown separately on ½ PDA at 25±2 °C for five to six days to encourage mycelial growth. Five to six fungal plugs taken from edge of the actively growing hyphae were then added to the cooled millet grains. The bottle was incubated at 25±2 °C and shaken daily for seven days to promote even distribution and uniform fungal colonization. After seven days of incubation, the fungal inoculum was grown on ½ PDA to determine the viability. The inoculated millet grains were randomly scattered on a ½ PDA plate and incubated for five to seven days at 25±2 °C to facilitate the fungal spore germination. After seven days plates were observed under the compound microscope for the mycelial growth around the millet grains. The prepared millet grain-fungal inoculum can be stored for up to 21 days at room temperature.

**Supplementary Section 2**

Experimental design of soil-free *in planta* pathogenicity assay

Development of Fusarium wilt disease rating scale, implementation and assessment

We observed disease symptom development in F5189 pathogen challenged seedlings and negative control seedlings from 18 lucerne cultivars (5 replicates each) daily for 14 days to develop a Fusarium wilt disease rating scale. The seedlings were rated at four time points: 3 , 6, 8 and 14 dpi within 14-day period on a scale from 0 to 6. The baseline for the disease rating is the colour differentiations observed at RSJs and the shoot axis up to cotyledons of the seedlings.

Supplementary Table S4. The Fusarium wilt disease scoring index used in soil free pathogenicity and bioprotection assays

| **Disease score** | **Description** |
| --- | --- |
| 0 | Green cotyledons/ healthy seedlings |
| 1 | Pinkish discolouration of the lower shoot |
| 2 | Reddish discolouration of the lower shoot |
| 3 | Reddish discolouration of the whole shoot |
| 4 | Wilting of the cotyledons |
| 5 | Reddish discolouration of the cotyledons |
| 6 | Death of the seedling |


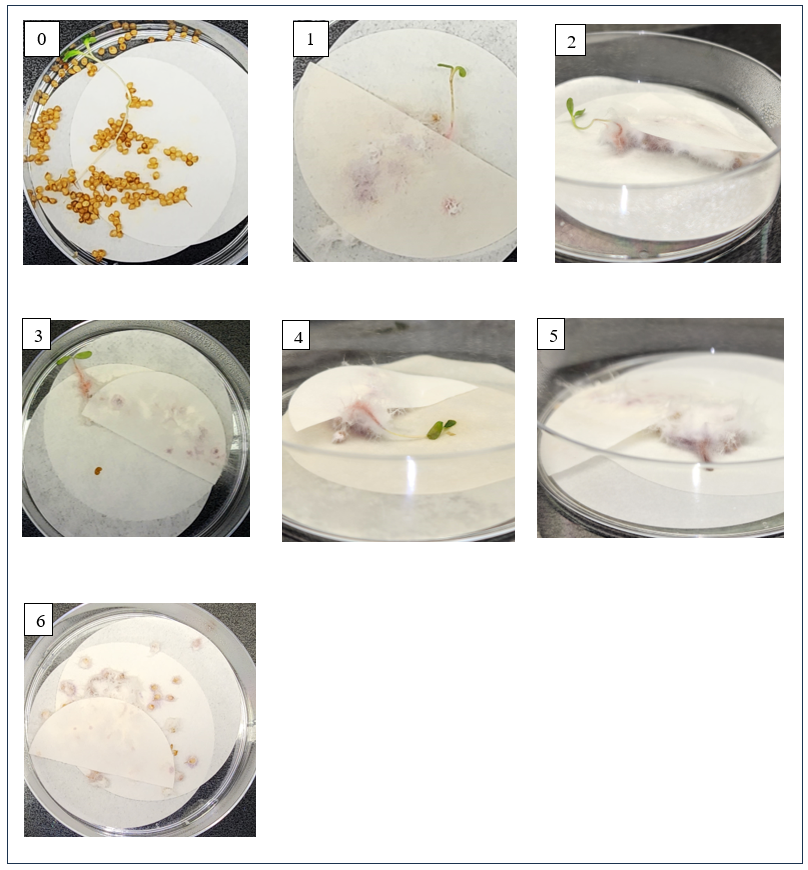


Supplementary Figure S3. Fusarium wilt disease progression with disease scoring index system used to assess pathogenicity assay. Soil free pathogenicity assay was set up in a standard petri dish.

**Supplementary section 3**

Experimental design of *in planta* pathogenicity assay in potting mix

Development of *Fusarium* wilt disease rating scale, implementation and assessment

We observed disease symptom development daily for 14 days to develop a *Fusarium* wilt disease rating scale. The seedlings were scored at five time points within 14-day period on a scale from 0 to 5. The baseline for the disease rating is the colour differentiations observed along the shoot axis up to leaves, wilting and stunting of the plants.

The two cultivars: Grazer and Sequel used in the soil-free *in planta* pathogenicity assay were tested for their disease susceptibility on soil. The 10 dap (days after planting) seedlings were transplanted into plug-shaped holes in pots lined with *Fusarium*-millet grain inoculum and were monitored for disease symptoms development for a period of 14 days to establish a disease score index system. According to the preliminary observations, the first visible symptom above ground was reddish-brownish discolouration of the cotyledons followed by yellowing of leaves progressing from ground level to the top resulting in wilting, leaf fall and stunting and ultimately dying.

Supplementary Table S8. The *Fusarium* wilt disease scoring index used in pathogenicity and bioprotection assays conducted in a potting mix based medium in growth cabinets.

| **Disease score** | **Description** |
| --- | --- |
| 0 | Green cotyledons/ healthy seedlings |
| 1 | Reddish discolouration of the cotyledons |
| 2 | Yellowing of the first true leaf |
| 3 | Yellowing of more than one leaf |
| 4 | Yellowing of all the leaves/ wilting/ stunting/ reddish-brownish discolouration of the basal stem |
| 5 | Death of the seedling |


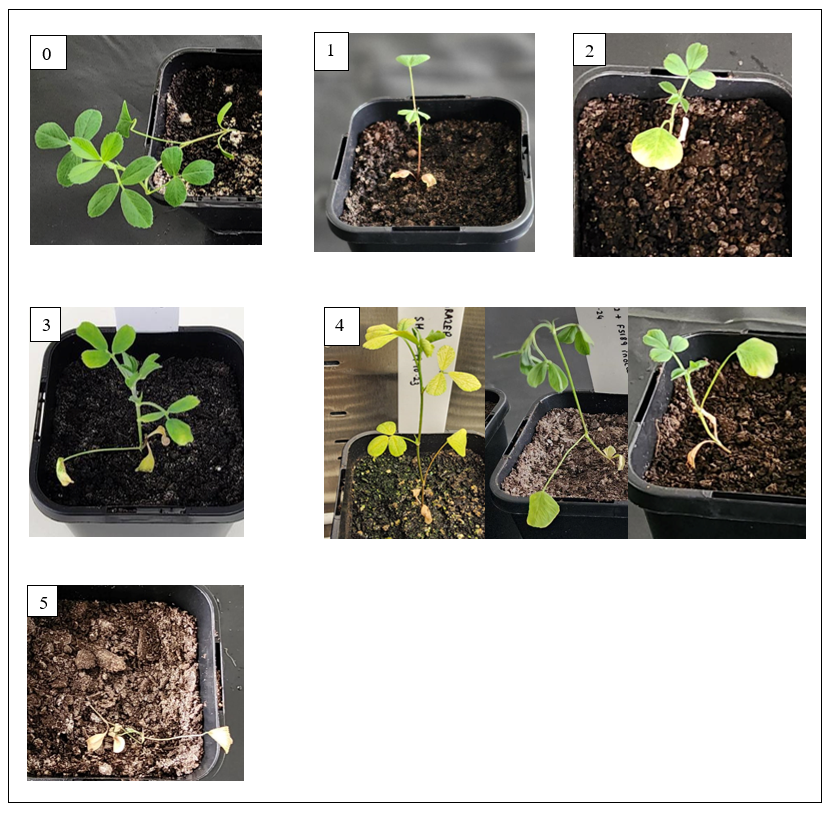


Supplementary Figure S4. Fusarium wilt disease progression with disease scoring index system used to assess pathogenicity and bioprotection assay. Both the assays were conducted in growth cabinets in a potting mix-based medium.

**References**

Herath D., S.S., Kaur, J., and Sawbridge, T. (2025). *Medicago* crop wild relative seed microbiome hosts a more multifaceted microbial community compared to domesticated lucerne (*Medicago sativa* L) of Australian origin Unpublished manuscript.
